# COVID-19 Policies, Pandemic Disruptions, and Changes in Child Mental Health and Sleep in the United States

**DOI:** 10.1001/jamanetworkopen.2023.2716

**Published:** 2023-03-13

**Authors:** Yunyu Xiao, Timothy T. Brown, Lonnie R. Snowden, Julian Chun-Chung Chow, J. John Mann

**Affiliations:** 1 Weill Cornell Medicine, NewYork Presbyterian, Department of Population Health Sciences, New York; 2 School of Public Health, University of California, Berkeley; 3 School of Social Welfare, University of California, Berkeley; 4 Departments of Psychiatry and Radiology, Columbia University Irving Medical Center, Columbia University, New York; 5 Division of Molecular Imaging and Neuropathology, New York State Psychiatric Institute, New York

## Abstract

**Importance:**

The adverse effects of COVID-19 containment policies disrupting child mental health and sleep have been debated. However, few current estimates correct biases of these potential effects.

**Objectives:**

To determine whether financial and school disruptions related to COVID-19 containment policies and unemployment rates were separately associated with perceived stress, sadness, positive affect, COVID-19–related worry, and sleep.

**Design, Setting, and Participants:**

This cohort study was based on the Adolescent Brain Cognitive Development Study COVID-19 Rapid Response Release and used data collected 5 times between May and December 2020. Indexes of state-level COVID-19 policies (restrictive, supportive) and county-level unemployment rates were used to plausibly address confounding biases through 2-stage limited information maximum likelihood instrumental variables analyses. Data from 6030 US children aged 10 to 13 years were included. Data analysis was conducted from May 2021 to January 2023.

**Exposures:**

Policy-induced financial disruptions (lost wages or work due to COVID-19 economic impact); policy-induced school disruptions (switches to online or partial in-person schooling).

**Main Outcomes and Measures:**

Perceived stress scale, National Institutes of Health (NIH)–Toolbox sadness, NIH-Toolbox positive affect, COVID-19–related worry, and sleep (latency, inertia, duration).

**Results:**

In this study, 6030 children were included in the mental health sample (weighted median [IQR] age, 13 [12-13] years; 2947 [48.9%] females, 273 [4.5%] Asian children, 461 [7.6%] Black children, 1167 [19.4%] Hispanic children, 3783 [62.7%] White children, 347 [5.7%] children of other or multiracial ethnicity). After imputing missing data, experiencing financial disruption was associated with a 205.2% [95% CI, 52.9%-509.0%] increase in stress, a 112.1% [95% CI, 22.2%-268.1%] increase in sadness, 32.9% [95% CI, 3.5%-53.4%] decrease in positive affect, and a 73.9 [95% CI, 13.2-134.7] percentage-point increase in moderate-to-extreme COVID-19–related worry. There was no association between school disruption and mental health. Neither school disruption nor financial disruption were associated with sleep.

**Conclusions and Relevance:**

To our knowledge, this study presents the first bias-corrected estimates linking COVID-19 policy-related financial disruptions with child mental health outcomes. School disruptions did not affect indices of children’s mental health. These findings suggest public policy should consider the economic impact on families due to pandemic containment measures, in part to protect child mental health until vaccines and antiviral drugs become available.

## Key Points

**Question:**

What are the separate bias-corrected associations of COVID-19-containment policy-related perceived school and financial disruptions with mental health and sleep in US children?

**Findings:**

In this nationwide cohort study of 6030 US children age 10 to 13 years, bias-corrected results from instrumental variable methods suggested that experiencing financial disruptions, despite supportive policies, was associated with a 205.2% increase in perceived stress, 112.1% increase in sadness, 32.9% decrease in positive affect, and a 73.9 percentage-point increase in COVID-19–related worry. School disruptions were not associated with changes in child mental health, and neither financial nor school disruptions were associated with sleep.

**Meaning:**

These findings suggest that public health authorities should consider the mental health impacts of pandemic-related disruptions when deciding mitigating policies.

## Teaser

This cohort study examines whether financial and school disruptions related to COVID-19 containment policies and unemployment rates were associated with perceived stress, sadness, positive affect, sleep, and COVID-19–related worry among US children.

## Introduction

More than 1 million people have died in the US from COVID-19.^1^ Pandemic-related containment policies, including school closures, social distancing, and restrictions of in-person activities, were essential to control infection before vaccination and antiviral medications became available.^2,3^ However, such policies may have unintended consequences for child mental health.^4,5,6^ It is important to evaluate psychological sequelae^2,3,7,8^ and sleep deterioration^9,10,11^ of pandemic-related economic crises^12^ and disrupted schooling^13,14^ on children.^15,16,17,18,19,20,21^ Yet, to date, rigorous quantitative information is lacking.

Although there have been efforts to evaluate COVID-19’s impact on child mental health,^22,23,24,25^ most have not adequately accounted for many simultaneously implemented COVID-19 policies.^26,27^ COVID-19 stringency and restrictive policies (eg, closures of schools and businesses) reduced viral spread and saved lives, but they also disrupted family finances (eg, via parental job losses or fewer working hours)^28,29,30,31^ and children’s schooling.^32,33^ Conversely, COVID-19 support-and-flexibility policies provided economic support (eg, income support, debt relief) to mitigate pandemic-related disruptions.^34,35^ The fact that these policies differed by targets, location, duration, and intensity^36,37^ makes it challenging to quantify how such heterogeneous policies impacted child mental health and sleep.^38,39,40^

Another limitation of existing literature includes the predominant use of cross-sectional studies and convenience samples,^18,23,37,41,42,43^ providing little causal-inferential evidence.^18,44,45,46^ Among the few studies accounting for confounding biases, most studied adults^47,48,49,50,51^ and COVID-19 transmission outcomes instead of mental health.^52,53,54,55^ One association study found that child stress increased when families experienced financial disruptions.^20^ Early evidence suggested that the pandemic's financial stress and school disruptions were associated with increased psychological stress and poorer adolescent sleep.^13,18,19,56,57,58,59^ However, bias-corrected estimates of the independent and separate associations of financial and school disruptions due to COVID-19 policies on child mental health and sleep have not been determined.^20^

The present study used instrumental variables (IV) analysis to disentangle the co-occurrences of school closures and financial disruptions and estimate independent, bias-corrected associations of school and financial disruptions with mental health and sleep. We hypothesized that policy-associated financial and school disruptions were associated with worse child mental health and sleep outcomes.

The Figure shows a directed acyclic graph^61^ to illustrate the hypothesized associations. Our approach was distinct from previous studies by simultaneously evaluating these 2 primary disruptions based on variations in dynamically changing policies and county-level economic conditions. We treated policies and county-level economic conditions as instruments. In our model, these instruments were related to child mental health and sleep only through school and financial disruptions, conditional on the inclusion of adequate controls, including COVID-19 incidence, children’s COVID-19-related media exposure, and socioeconomic covariates.

**Figure.**
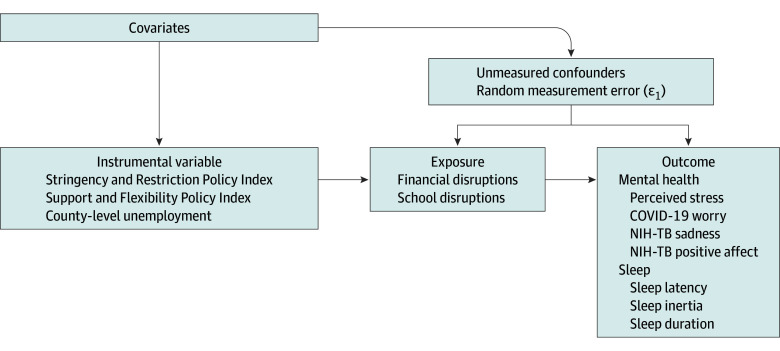
Study Design, Data Sources, and Variable Measures Abbrevation: NIH–TB, National Institutes of Health–Toolbox. Instrumental variables analyses incorporate the use of exogenous external factors (external factors whose values cannot be affected by the individual children and parents being examined) whose impact on outcomes only works through the endogenous exposures of interest (family financial disruption and school disruptions), conditional on the included covariates.^60^ Detailed explanations of our instruments’ validity and strength can be found in eMethods in Supplement 1. Graphic schema of the study timeline is available in the eFigure in Supplement 1.

## Methods

This cohort study followed the Strengthening the Reporting of Observational Studies in Epidemiology (STROBE) reporting guideline. Centralized institutional review board (IRB) approval was obtained from the University of California, San Diego. This project was exempt from IRB approval after we obtained the Data Use Certificate from the National Institute of Mental Health Data Archive. Parents or guardians provided written informed consent, and children provided verbal assent.

### Study Design and Participants

We used the Adolescent Brain Cognitive Development (ABCD) Study,^19^ a longitudinal stratified probability sample of public and private schools from 21 US metropolitan areas containing 11 878 children aged 9 to 10 years from 2016 to 2018 (baseline). All schools within 50 miles of study sites were approached. Schools with more than 10% Black children were oversampled by around 50%. The age, sex, and race and ethnic composition of the sample was designed to match closely with the American Community Survey (ACS) and represent more than 20% of the US population in this age group.^62^

Our analytic sample was from the main sample and COVID Rapid Response Research (RRR) First and Second Data Releases (ABCD 4.0 Release).^63,64^ ABCD sent 6 online surveys to children and parents or guardians from May 2020 to May 2021 (eFigure in Supplement 1). Children and parents or guardians could return the survey at any time. Some survey responses were incomplete, with some differences between survey responders and nonresponders (eTable 3 in Supplement 1). Details of the ABCD study are available in eMethods in Supplement 1 and elsewhere.^20,21,22,23^ Eligibility criteria for study inclusion were any survey fielded and returned in 2020, with available sociodemographics. After imputing missing values of covariates (not including outcomes), our imputed analytic sample is an unbalanced panel containing 25 555 observations of 8400 children (mental health) and 25 948 observations of 8472 children (sleep).

We linked ABCD data to 3 external data sets: (1) US state-level policy data from the Oxford COVID-19 Government Response Tracker (OxCGRT),^65^ which includes policy indexes constructed from COVID-19-related policies (eMethods in Supplement 1)^37^; (2) county-level COVID-19 incidence derived from John Hopkins University COVID-19 data^24^; and (3) county-level monthly unemployment rates from the US Bureau of Labor Statistics (BLS).^26^ These external data sets were geocoded per participant on the date that the survey was disseminated.^64^ Survey 6 was omitted because 2021 unemployment rates linked to the ABCD data were unavailable during manuscript preparation.

### Measures

#### Outcomes

We included 4 mental health outcomes (perceived stress, sadness, positive affect, COVID-19-related worry) and 3 sleep outcomes (abnormal lengths of inertia, latency, duration) (eMethods in Supplement 1). Perceived stress was measured with the 4-item Perceived Stress Scale (PSS-4).^28^ Children were asked how often in the last month they experienced stressful situations (eg, have you felt that things were going your way?) using a 5-point scale (from 0 = never to 4 = very often). Higher summed values represent higher stress. PSS-4 has been used to assess mental health during COVID-19 and showed high reliability or validity.^46,66,67^

Sadness (8 items; eg, feeling lonely) and positive affect (9 items; eg, feeling happiness) were measured using the National Institutes of Health Toolbox Emotion Battery (NIH TB-EB) on a 5-point scale (from 1 = never to 5 = almost always). We followed NIH Toolbox guidelines^68^ to compute composite scores. Prior studies showed high reliability or validity.^69,70,71^

COVID-related worry was measured by asking children how worried they have been about COVID-19 (1 = not at all; 2 = slightly; 3 = moderately; 4 = very; 5 = extremely). We coded it as binary (0 = not at all/slightly, 1 = moderately to extremely) as in our prior study.^20^

Sleep was measured by the Munich Chronotype Questionnaire (MCTQ),^72^ a previously validated survey^73^ asking past-week sleep timing from bedtime to wake-up time. ABCD-COVID-19-RRR did not differentiate school and free days. We calculated dichotomized measures for sleep latency (time to fall asleep),^74,75,76^ inertia (time drowsy after awakening),^77,78^ and duration^79,80^ (0 = reference range, 1 = outside reference range).

#### Exposures

School disruption was a binary variable indicating whether children received remote schooling or attended fewer days weekly. Financial disruption was a binary variable answered by parents in each survey: “since January 2020, has anyone in your household lost wage, sales, or work due to the impact of coronavirus on employment, business, or the economy?”

#### Instrumental Variables

We used 3 instruments. The first 2 came from OxCGRT,^81^ measuring time-varying policy intensity by interview dates. OxCGRT has appeared in multiple studies (eMethods and eTable 1 in Supplement 1).^82,83,84,85^ To distinguish policy purposes, we recategorized OxCGRT components into 2 new indexes: the Stringency and Restrictive Policy Index (SRPI) and the Support and Flexibility Policy Index (SFPI).

The SRPI reflected 9 policies to control transmission through restricting travel and in-person business or schooling, including school closure, workplace closure, canceling public events, gathering restrictions, public transport closure, local movement restrictions, international travel restrictions, and information campaigns. The SFPI reflected 7 policies to alleviate economic hardship and improve health using approaches not restricting travel. It includes income support, debt relief, testing, contact tracing, masking, vaccination, and older adult protection. The values of SRPI and SFPI changed over time as policies within each subcategory changed dynamically. We logarithmically transformed SRPI and SFPI to account for diminishing returns. The third instrument was county-level monthly BLS 2020 unemployment rates.

#### Covariates

We included baseline sociodemographics, including children’s age, biological sex, race or ethnicity (ie, Asian, Black, Hispanic, other or multiracial, and White), parental education (Bachelor’s degree or higher or no degree), parental marital status (married or not married), and household income (≥$100 000/y or not). Other or multiracial children were American Indian/Native American or Alaska Native, Native Hawaiian, Guamanian, and other races. Considering race and ethnicity can help to reduce bias due to potential confounding factors. We adjusted for county-level COVID-19 incidence per 100 000 hours children were exposed to COVID-19-related news using the question, “over the past week, how much time per day do you think your child has been getting news from television news sources about the coronavirus and its impact?” (eTable 2 in Supplement 1).

### Statistical Analysis

We used IV analysis to exploit the natural experiment^86^ where COVID-19 policies and unemployment rates varied across time and geography.^87^ IV analysis exploits exogenous factors (ie, factors whose values cannot be affected by individual children or parents) that are correlated with exposures but not second-stage error terms. In other words, the IV model assumes that instruments are exogenous, strong, and only associated with outcomes through school and financial disruptions, conditional on covariates.^60^ Additionally, the model assumed that instruments have monotonic associations with exposures (eMethods in Supplement 1).

The SFPI and SRPI were necessarily exogenous to individual children’s mental health and sleep. However, policies captured by SFPI and SRFI may associate with child well-being through families’ and friends’ COVID-19 experiences. Therefore, we included COVID-19 incidence as a covariate. Additionally, children were unlikely to be aware of SFPI and SRPI policies or unemployment rates, except via media; thus we included COVID-19 media exposure as a covariate.

County-level unemployment rates^88^ reflected industrial composition and labor-market participation in local areas, which were necessarily exogenous to child mental health and sleep because the labor market in each site was too large to be affected by any family’s unemployment. Thus, conditional on controlling for sociodemographics, COVID-19 incidence, and children’s media exposure, our instruments should only associate with children’s mental health and sleep through policy-induced financial and school disruptions. We did not control variables on the pathway from school or financial disruption to child mental health and sleep, such as social isolation, lacking routines, sedentary behaviors, and parental stress. While understanding such pathways is important, it was beyond our study scope. Lastly, children were often not free of stress or had low stress at baseline.

To implement IV, we regressed our instruments and covariates against each exposure (2 separate first-stage equations for each model). We then took the projected values of the 2 exposures from the first-stage equations and regressed these (along with the same covariates) against each outcome. We used 2-stage limited information maximum likelihood (2SLIML) regression, which is approximately median unbiased,^89,90^ provided finite-sample bias reduction, and the same asymptotic distribution as 2-stage least squares allowing for the use of smaller critical values when applying Stock-Yogo weak instrument tests.^91^ The Kleibergen-Papp Wald rk *F* statistic was calculated to check the strength of the instrument set. We described our 2SLIML models further in eMethods in Supplement 1.

We estimated logarithmic-linear models for continuous outcomes (perceived stress, sadness, positive affect). The coefficients of these equations were transformed ([exp(coefficient) − 1] × 100).^92^ The transformed coefficients thus represented the change in the percentage of the outcome when a binary variable changes from 0 to 1 (eg, a change from no financial disruptions [0] to financial disruption [1] results in a 202.2% change in perceived stress) (Table 1).

**Table 1.** Associations of Financial and School Disruptions With Mental Health Outcomes^a^ (Missing Values Imputed)

Characteristics	Perceived stress (natural log), % (95% CI)	COVID-19 worry (binary)y, percentage points (95% CI)		Sadness (natural log), % (95% CI)	Positive qffect (natural log), % (95% CI)
Key exposures					
Financial disruptions (binary)^b^	205.155 (52.904 to 509.007)^c^		73.946 (13.168 to 134.725)^d^	112.056 (22.164 to 268.093)^c^	−32.904 (−53.371 to −3.452)^d^
School disruptions (binary)^e^	−8.101 (−36.896 to 33.834)		2.048 (−18.763 to 22.860)	−20.292 (−38.891 to 3.969)	−4.075 (−16.883 to 10.706)
Child sociodemographic characteristics					
Age, y	4.952 (2.981 to 6.961)^f^		1.986 (0.310 to 3.662)^f^	3.976 (2.535 to 5.437)^f^	−2.188 (−3.205 to −1.160)^f^
Female	12.473 (8.733 to 16.341)^f^		8.789 (6.138 to 11.440)^f^	16.950 (13.718 to 20.274)^f^	−4.180 (−5.908 to −2.420)^f^
Asian	6.899 (−3.595 to 18.535)		16.636 (9.282 to 23.991)^f^	4.801 (−1.618 to 11.638)	−0.368 (−5.068 to 4.566)
Black	3.950 (−4.910 to 13.636)		16.916 (9.978 to 23.853)^f^	−7.558 (−12.774 to −2.029)^c^	5.615 (0.971 to 10.473)^f^
Hispanic	1.111 (−3.703 to 6.167)		7.482 (2.826 to 12.139)^c^	−1.478 (−6.013 to 3.277)	2.602 (−1.072 to 6.412)
Other race	3.436 (−3.447 to 10.809)		5.088 (−4.730 to 14.906)	−0.022 (−6.328 to 6.708)	0.180 (−2.680 to 3.124)
Parental characteristics					
≥Bachelor’s degree	10.854 (−0.154 to 23.075)		12.156 (2.227 to 22.085)^d^	10.086 (1.497 to 19.401)^d^	−3.566 (−8.703 to 1.860)
<$100 000 per year	−3.324 (−9.532 to 3.310)		−7.281 (−12.708 to −1.855)^c^	−2.294 (−6.349 to 1.937)	2.133 (−1.606 to 6.014)
Not married	7.562 (1.476 to 14.012)^d^		4.036 (0.443 to 7.629)^d^	6.014 (2.168 to 10.005)^c^	−4.625 (−7.480 to −1.682)^c^
Environmental factors (COVID-19 related)					
Child exposure to media, h	0.276 (−0.101 to 0.655)		−0.055 (−0.223 to 0.114)	0.115 (−0.154 to 0.386)	−0.204 (−0.395 to −0.013)^d^
COVID-19 incidence/100 000 population	0.017 (−0.044 to 0.078)		0.019 (−0.028 to 0.066)	−0.012 (−0.069 to 0.046)	0.014 (−0.039 to 0.066)
Longitudinal survey indicators					
Survey 2	−8.617 (−25.010 to 11.359)		−0.107 (−11.398 to 11.184)	NA	1.398 (−5.322 to 8.596)
Survey 3	−11.585 (−28.677 to 9.604)		−0.868 (−12.437 to 10.701)	−14.406 (−26.683 to −0.072)^d^	NA
Survey 4	1.264 (−1.823 to 4.448)		−0.001 (−2.045 to 2.044)		NA
Survey 5	1.345 (−2.928 to 5.806)		−2.018 (−5.177 to 1.141)	2.668 (0.421 to 4.965)^d^	NA
Constant	71.833 (16.484 to 153.481)^c^		−33.456 (−72.589 to 5.676)	511.731 (337.245 to 755.846)^f^	5579.676 (4243.614 to 7326.700)^f^
Weak instrument test for LIML (joint test of both first-stage results)					
Kleibergen-Paap rk Wald *F* statistic	10.49		10.49	6.54	10.08
Stock-Yogo LIML critical value (10%)	5.44		5.44	5.44	5.44
Observations (individuals)	25 555 (8400)		25 555 (8400)	14 610 (7447)	9408 (6302)

Abbreviations: LIML, limited information maximum likelihood; NA, not applicable.

^a^ Missing data in the unbalanced panel was imputed using hot deck imputation. Graphic schema of the study timeline is available in the eFigure in Supplement 1.

^b^ Financial disruptions indicate whether, since January 2020, anyone in the household lost wages, sales, or work due to the impact of the coronavirus on employment, business, or the economy (binary).

^c^ *P* ≤.01.

^d^ *P* ≤.05.

^e^ School disruptions are measured by self-report levels of online or partial in-person instructional settings (binary).

^f^ *P* ≤.001.

For dichotomous outcomes, we used linear probability models (LPM) for the following reasons. First, all binary dependent variables are in the range (20% to 80%), where LPM performs virtually identical to marginal estimates from logistic or probit regression models.^93^ Second, our sample size was large, making our assumption about asymptotic consistency reasonable.^91^ Third, IV logistic regression models are not technically appropriate when there are multiple binary endogenous variables, whereas IV LPM is technically appropriate.^94^ Finally, LPM has long appeared in the medical literature.^95,96,97^ Coefficients transformed (multiplied by 100) represent marginal percentage point changes.

Our models accounted for missing covariate data using hot deck imputation to maintain a larger sample size, and we also performed multiple imputations as a sensitivity check.^98,99^ All analyses were performed using Stata version 16 (StataCorp), incorporated ACS-based probability weights, and site-level clustered standard errors.^100,101^ Data analysis was conducted from May 2021 to January 2023. Two-tailed tests were conducted, and *P* ≤ .05 was considered statistically significant. 

## Results

### Descriptive Characteristics

This study included 6030 unique individuals in the mental health sample (weighted median [IQR] age, 13 [12-13] years; 2947 females [48.9%]; 273 Asian children [4.5%]; 461 Black children [7.6%]; 1167 Hispanic children [19.4%], 3783 White children [62.7%], and 347 children of other or multiracial ethnicity [5.7%]) (eTable 4 in Supplement 1). It included 6080 individual children in the sleep sample (weighted median [IQR] age, 13 [12-13] years; 2988 females [48.8%]; 273 Asian children [4.5%]; 470 Black children [7.7%]; 1174 Hispanic children [19.3%], 3814 White children [62.7%], and 350 children of other or multiracial ethnicity [5.8%]) (eTable 4 in Supplement 1). Table 2 and Table 3 show unimputed, descriptive statistics per survey (survey columns) and overall unbalanced panel totals (columns containing repeated observations).

**Table 2.** Descriptive Statistics of Mental Health Sample (Unimputed Data)^a^

Characteristics	No. (%)					
	Survey 1 (n = 4008)	Survey 2 (n = 4030)	Survey 3 (n = 3870)	Survey 4 (n = 3626)	Survey 5 (n = 2757)	Total (N = 6030)^b^
Sociodemographic characteristics						
Age, median (IQR), y	12 (12-13)	13 (12-13)	13 (12-13)	13 (12-14)	13 (12-14)	13 (12-13)
Sex at birth						
Female	2003 (50.0)	1990 (49.4)	1905 (49.2)	1824 (50.3)	1419 (51.4)	9141 (50.0)
Male	2005 (50.0)	2040 (50.6)	1965 (50.8)	1801 (49.7)	1340 (48.6)	9150 (50.0)
Race and ethnicity						
Asian	207 (5.2)	208 (5.2)	210 (5.4)	199 (5.5)	157 (5.7)	982 (5.4)
Black	224 (5.6)	272 (6.8)	242 (6.3)	228 (6.3)	170 (6.2)	1136 (6.2)
Hispanic	742 (18.5)	722 (17.9)	680 (17.6)	628 (17.3)	444 (16.1)	3215 (17.6)
Other race^c^	223 (5.6)	228 (5.7)	216 (5.6)	214 (5.9)	139 (5.0)	1021 (5.6)
White	2612 (65.2)	2599 (64.5)	2521 (65.2)	2357 (65.0)	1849 (67.0)	11 938 (65.3)
Parental education						
<Bachelor	2784 (69.5)	2734 (67.8)	2647 (68.4)	2500 (69.0)	1939 (70.3)	12 603 (68.9)
≥Bachelor	1224 (30.5)	1296 (32.2)	1223 (31.6)	1125 (31.0)	819 (29.7)	5688 (31.1)
Household income, $ per year						
<50 000 or 50 000-100 000	2458 (61.3)	2552 (63.3)	2425 (62.7)	2286 (63.0)	1720 (62.4)	11 441 (62.5)
>100 000	1550 (38.7)	1478 (36.7)	1445 (37.3)	1340 (37.0)	1038 (37.6)	6850 (37.5)
Parental marital status						
Not married	1005 (25.1)	1079 (26.8)	1015 (26.2)	978 (27.0)	697 (25.3)	4774 (26.1)
Married	3003 (74.9)	2951 (73.2)	2854 (73.8)	2648 (73.0)	2062 (74.7)	13 517 (73.9)
Child exposure to media, mean (SD), h	0.8 (3.8)	1.0 (4.2)	1.3 (4.8)	1.4 (4.9)	1.5 (5.4)	1.2 (4.6)
COVID-19 incidence/100 000, median (IQR)	5 (1-10)	6 (3-13)	12 (5-17)	10 (5-18)	70 (41-93)	9 (3-20)
Outcomes, median (IQR)						
Perceived stress (range: 0-16)	5 (3-8)	5 (3-7)	5 (3-7)	5 (3-8)	6 (4-8)	5 (3-7)
Sadness (range: 8-40)	13 (9-19)	NA	12 (9-18)	NA	14 (9-20)	13 (9-19)
Positive affect (range: 9-45)	NA	35 (29-41)	NA	35 (27-41)	NA	35 (27-41)
COVID-19 related worry						
Not at all/slightly	2361 (58.9)	2415 (59.9)	2432 (62.8)	2213 (61.1)	1669 (60.5)	11091 (60.6)
Moderately/very/extremely	1647 (41.1)	1614 (40.1)	1438 (37.2)	1412 (38.9)	1089 (39.5)	7199 (39.4)
Key exposures						
Financial disruptions [range: 0-4], mean (SD)						
No change	2113 (52.7)	2100 (52.1)	2105 (54.4)	2035 (56.1)	1509 (54.7)	9862 (53.9)
Experienced disruptions	1895 (47.3)	1930 (47.9)	1765 (45.6)	1590 (43.9)	1249 (45.3)	8429 (46.1)
School disruptions						
No change	496 (12.4)	2648 (65.7)	2875 (74.3)	804 (22.2)	573 (20.8)	7396 (40.4)
Experienced disruptions	3512 (87.6)	1382 (34.3)	995 (25.7)	2822 (77.8)	2185 (79.2)	10895 (59.6)
Instrumental variables, median (IQR)						
SRPI	68 (63-74)	61 (55-68)	61 (57-66)	53 (47-62)	62 (49-69)	61 (54-68)
SFPI	15 (13-16)	16 (13-17)	12 (10-17)	11 (9-15)	12 (9-16)	13 (11-16)
BLS unemployment rate	11 (10-13)	10 (8-12)	7 (6-9)	6 (4-7)	6 (4-7)	8 (6-10)

Abbreviations: BLS, Bureau of Labor Statistics; NA, not applicable; SFPI, Support and Flexibility Policy Index; SRPI, Stringency and Restriction Policy Index.

^a^ Unbalanced Longitudinal Panel: Each survey includes the total number of individuals with complete cases before hot deck imputation.

^b^ Includes 6030 individuals and 18 291 individual-interview date observations.

^c^ Other or multiracial children were American Indian/Native American or Alaska Native, Native Hawaiian, Guamanian, and other races.

**Table 3.** Descriptive Statistics for Sleep Sample (Unimputed Data)^a^

Characteristics	No. (%)					
	Survey 1 (n = 4051 individuals)	Survey 2 (n = 4076 individuals)	Survey 3 (n = 3920 individuals)	Survey 4 (n = 3704 individuals)	Survey 5 (n = 2816 individuals)	Total (n = 6080)^b^
Sociodemographic characteristics						
Age, median (IQR), y	12 (12-13)	13 (12-13)	13 (12-13)	13 (12-14)	13 (12-14)	13 (12-13)
Sex at birth						
Female	2023 (49.9)	2013 (49.4)	1930 (49.2)	1858 (50.2)	1446 (51.4)	9270 (49.9)
Male	2027 (50.1)	2062 (50.6)	1990 (50.8)	1846 (49.8)	1370 (48.6)	9296 (50.1)
Race and ethnicity						
Asian	208 (5.1)	209 (5.1)	212 (5.4)	205 (5.5)	158 (5.6)	992 (5.3)
Black	224 (5.5)	277 (6.8)	247 (6.3)	235 (6.3)	175 (6.2)	1158 (6.2)
Hispanic	753 (18.6)	732 (18.0)	692 (17.7)	636 (17.2)	454 (16.1)	3267 (17.6)
Other race^c^	225 (5.6)	231 (5.7)	220 (5.6)	219 (5.9)	139 (5.0)	1035 (5.6)
White	2639 (65.2)	2626 (64.4)	2548 (65.0)	2410 (65.1)	1890 (67.1)	12 114 (65.2)
Parental education						
<Bachelor’s degree	2807 (69.3)	2761 (67.8)	2673 (68.2)	2553 (68.9)	1978 (70.3)	12 772 (68.8)
≥Bachelor’s degree	1244 (30.7)	1314 (32.2)	1247 (31.8)	1151 (31.1)	837 (29.7)	5794 (31.2)
Household income, $ per year						
<50 000 or 50 000-100 000	2484 (61.3)	2582 (63.4)	2466 (62.9)	2330 (62.9)	1753 (62.2)	11 615 (62.6)
>100 000	1566 (38.7)	1493 (36.6)	1454 (37.1)	1374 (37.1)	1063 (37.8)	6951 (37.4)
Parental marital status						
Not married	1018 (25.1)	1094 (26.9)	1033 (26.4)	996 (26.9)	711 (25.2)	4853 (26.1)
Married	3032 (74.9)	2981 (73.1)	2887 (73.6)	2708 (73.1)	2105 (74.8)	13 713 (73.9)
Child exposure to media, mean (SD), h	0.8 (3.8)	1.0 (4.2)	1.3 (4.8)	1.3 (4.9)	1.5 (5.4)	1.2 (4.6)
COVID-19 Incidence/100 000 population, median (IQR)	5 (1-10)	6 (3-13)	12 (5-17)	10 (5-17)	70 (41-92)	9 (3-20)
Outcomes						
Sleep latency						
Reference range	972 (24.0)	1004 (24.6)	936 (23.9)	928 (25.1)	674 (24.0)	4514 (24.3)
Outside of reference range	3078 (76.0)	3072 (75.4)	2984 (76.1)	2776 (74.9)	2141 (76.0)	14 052 (75.7)
Sleep inertia						
Reference range	969 (23.9)	1025 (25.2)	933 (23.8)	756 (20.4)	531 (18.9)	4215 (22.7)
Outside of reference range	3081 (76.1)	3050 (74.8)	2987 (76.2)	2948 (79.6)	2285 (81.1)	14351 (77.3)
Sleep duration						
Reference range	2109 (52.1)	2269 (55.7)	2154 (55.0)	1156 (31.2)	1095 (38.9)	8783 (47.3)
Outside of reference range	1942 (47.9)	1807 (44.3)	1766 (45.0)	2548 (68.8)	1720 (61.1)	9783 (52.7)
Key exposures						
Financial disruption						
No change	2128 (52.5)	2121 (52.0)	2126 (54.2)	2088 (56.4)	1546 (54.9)	10 008 (53.9)
Experienced disruption	1923 (47.5)	1955 (48.0)	1794 (45.8)	1616 (43.6)	1270 (45.1)	8558 (46.1)
School disruption						
No change	505 (12.5)	2675 (65.6)	2905 (74.1)	818 (22.1)	591 (21.0)	7494 (40.4)
Experienced disruption	3545 (87.5)	1401 (34.4)	1015 (25.9)	2886 (77.9)	2225 (79.0)	11 072 (59.6)
Instrumental variables, median (IQR)						
SRPI	68 (63-74)	61 (55-69)	61 (57-66)	53 (47-62)	62 (49-69)	61 (54-68)
SFPI	15 (13-16)	16 (13-17)	12 (10-17)	11 (9-15)	12 (9-16)	13 (11-16)
BLS unemployment rate	11 (10-13)	10 (8-12)	7 (6-9)	6 (4-7)	6 (4-7)	8 (6-10)

Abbreviations: BLS, Bureau of Labor Statistics; SFPI, Support and Flexibility Policy Index; SRPI, Stringency and Restriction Policy Index.

^a^ Unbalanced longitudinal panel: each survey includes the total number of individuals with complete cases before hot deck imputation.

^b^ Includes 6080 individuals (18 566 individual-interview date observations).

^c^ Other or multiracial children were American Indian/Native American or Alaska Native, Native Hawaiian, Guamanian, and other races.

Sociodemographics in our study sample differed from the general population.^102,103^ Compared with individuals who responded to the survey, people who did not respond were more likely to be male, Black, Hispanic, or other race; had unmarried parents; parents with less than a Bachelor’s degree; and were low-to-middle income (eTable 3 in Supplement 1).

### Associations Between COVID-19 Disruptions and Child Mental Health and Sleep

Using our imputed analytic data sets, our instruments sets were sufficiently strong (Stock-Yogo weak instrument tests) (eTables 3 and 4 in Supplement 1).^104^ In all cases, the Kleibergen-Paap Wald rk *F* statistic of the joint strength of the complete set of instruments was equal to or larger than the critical value for 10% maximal LIML size.

Children with family financial disruptions experienced a mean 205.2% (95% CI, 57.9%-509.0%) increase in perceived stress, a mean 73.9 (95% CI, 14.22-48.45) percentage-point increase in moderate-to-extreme COVID-19–related worry, a mean 112.1% (95% CI, 22.2%-268.1%) increase in sadness, and a mean 32.9% (95% CI, 3.5%-53.4%) decrease in positive affect. There was no significant association between school disruptions and mental health. Neither family financial disruptions nor school disruptions were associated with children’s sleep (Table 4). Coefficient estimates were not statistically different when multiple imputation rather than hot deck imputation was used. eTables 5 and 6 in Supplement 1 show the full first-stage results corresponding to the second-stage results presented in Table 3 and Table 4, respectively.

**Table 4.** Association of Financial and School Disruptions With Sleep (Missing Values Imputed)^a^

Characteristics	Second stage transformed coefficient, percentage point (robust 95% CI)		
	Abnormal sleep latency^b^	Abnormal sleep inertia^c^	Abnormal sleep duration^d^
Key exposures			
Financial disruptions (binary)	10.279 (−11.775 to 32.332)	−10.848 (−46.480 to 24.784)	−11.046 (−61.223 to 39.131)
School disruptions (binary)	−12.773 (−26.022 to 0.476)	−12.423 (−29.259 to 4.413)	−2.460 (−20.144 to 15.223)
Child sociodemographic characteristics			
Age, y	0.096 (−0.537 to 0.729)	−2.426 (−3.620 to −1.232)^e^	5.527 (4.214 to 6.841)^e^
Female	−1.246 (−3.253 to 0.761)	−5.721 (−7.121 to −4.321)^e^	0.955 (−0.306 to 2.216)
Asian	4.385 (0.159 to 8.611)^f^	0.079 (−7.120 to 7.277)	−5.989 (−12.301 to 0.324)
Black	3.348 (−0.166 to 6.862)	2.185 (−3.021 to 7.391)	6.872 (1.145 to 12.598)^f^
Hispanic	4.340 (1.644 to 7.035)^g^	2.493 (−0.367 to 5.353)	0.745 (−2.609 to 4.099)
Other race^h^	1.099 (−2.229 to 4.428)	0.686 (−2.937 to 4.309)	3.919 (−0.195 to 8.033)
Parental characteristics			
≥Bachelor’s degree	0.287 (−2.684 to 3.258)	−2.201 (−7.049 to 2.646)	−0.027 (−6.845 to 6.790)
<$100 000 per year	0.693 (−1.662 to 3.049)	0.158 (−2.857 to 3.173)	0.666 (−2.910 to 4.243)
Not married	1.606 (−0.294 to 3.507)	−1.285 (−4.230 to 1.661)	1.777 (−1.264 to 4.817)
Environmental factors (COVID-19 related)			
Child exposure to media, h	0.098 (−0.008 to 0.203)	0.011 (−0.126 to 0.149)	0.048 (−0.098 to 0.195)
COVID-19 incidence/100 000 population	0.016 (−0.009 to 0.040)	0.016 (−0.016 to 0.048)	0.003 (−0.035 to 0.042)
Longitudinal survey indicators			
Survey 2	−7.316 (−14.411 to −0.220)^f^	−7.394 (−16.106 to 1.317)	−3.438 (−12.487 to 5.612)
Survey 3	−7.737 (−15.071 to −0.404)^f^	−6.975 (−16.531 to 2.581)	−4.705 (−14.193 to 4.783)
Survey 4	−2.061 (−3.911 to −0.210)^f^	2.598 (0.904 to 4.292)^g^	17.611 (14.459 to 20.762)^e^
Survey 5	−1.598 (−3.577 to 0.381)	3.395 (1.343 to 5.448)^g^	9.912 (6.925 to 12.899)^e^
Constant	79.294 (62.225 to 96.362)^e^	126.289 (106.810 to 145.768)^e^	−15.814 (−48.247 to 16.618)
Weak instrument test for LIML (joint test of both first-stage results)			
Kleibergen-Paap rk Wald F statistic	11.32	11.32	11.32
Stock-Yogo LIML critical value (10%)	5.44	5.44	5.44
Observations (individuals)	25 948 (8472)	25 948 (8472)	25 948 (8472)

Abbreviation: LIML, limited information maximum likelihood.

^a^ Missing data in the unbalanced panel was imputed using hot deck imputation. Graphic schema of the study timeline is available in the eFigure in Supplement 1.

^b^ Sleep latency (mins) was the time from turning the light off to falling asleep, and we calculated pathological/abnormal sleep latency, which is a binary variable categorizing child whose sleep latency is outside of the reference range between 10 to 26 minutes, was calculated.

^c^ Sleep inertia (mins) was the time taken to get out of bed, and abnormal sleep inertia, which is a binary variable categorizing child whose sleep inertia is outside of the reference range between 15 to 30 minutes, was calculated.

^d^ Sleep duration (hours) was first calculated using sleep end subtracted by sleep onset, and abnormal sleep duration, a binary variable categorizing children with insufficient sleep duration under 8 hours, was calculated.

^e^ *P* ≤.001.

^f^ *P* ≤.05.

^g^ *P* ≤.01.

^h^ Other or multiracial children were American Indian/Native American or Alaska Native, Native Hawaiian, Guamanian, and other races.

## Discussion

To our knowledge, this study provides the first instrumental variables-based evidence that family financial disruptions, despite supportive policies, are associated with greater perceived stress, sadness, and COVID-19–related worry and less positive affect in children. Contrary to our hypothesis, school disruptions were not associated with mental health or abnormal sleep, and financial disruptions were not associated with abnormal sleep.

During the pandemic, policy makers faced the tradeoff between reducing COVID-19 infection rates and causing potentially negative economic and mental health impacts when implementing SRPI policies.^105^ Prior cross-sectional studies found that COVID-19-related stressors were associated with psychological stress among children and adolescents.^56,57^ Consistent with a prior longitudinal study showing greater increases in trajectories of perceived stress and COVID-19 worry among children experiencing parental unemployment,^20^ we found that the impact of COVID-19 policies on child mental health was primarily through family financial disruption.^20,22,106,107^ Families play an important role in child mental health, and family resilience was more critical during the pandemic.^20,108^ Since greater psychological distress may have spillover impacts on learning loss, quality of family and peer relationships, and severe mental illness,^109,110,111^ addressing family financial stress may reduce the negative impact on child development in the future recovery from long COVID-19.

Contrary to our hypothesis and previous research findings that COVID-19 disruptions worsened sleep patterns, we found no association between economic or school disruption with abnormal sleep.^13,58,112,113^ This is contrary to studies in Switzerland,^13^ Singapore,^45,58^ and UK^112^ that found children and adolescents slept longer during COVID-19. We attribute this to different study designs and nation-specific contexts.

Our results call for clinicians to assess financial disruptions when supporting child mental health.^22,114^ We provide evidence that optimized infection mitigation policies during the pandemic could prioritize child mental health principally via economic relief to families. Strategic use of risk-mitigation policies may lower continuing societal costs.^105,115^

The current study’s strengths include being the first to use large, population-based, geocoded, longitudinal data to measure child mental health outcomes during the COVID-19 pandemic. Building upon the literature,^13,56,57,59^ our instrumental variable method corrected bias due to confounding, measurement error, and reverse causation, but only to the extent that the assumptions underlying the IV model hold.^116^ The IV model in this study is designed to attribute variation in child mental health and sleep outcomes to school and family financial disruptions, where such disruptions only vary to the degree they are associated with dynamically changing exogenous policies (SRPI and SFPI indexes) and county-level unemployment rates.

### Limitations

This study has limitations. First, while our IV method corrected bias due to confounding, measurement error, and reverse causation to the extent that the underlying IV assumptions hold,^116^ any bias-correction occurring due to the IV model is limited to the parameters for family financial and school disruptions and does not extend to control variable parameters. Second, ABCD measures are self-reported. While the scales for perceived stress, sadness, positive affect, and sleep are validated, responses may be subject to social desirability and recall biases. We also assessed sleep schedule instead of sleep quality.^112^ The ABCD-COVID period surveys did not include the severity or determination of new psychiatric disorder onset. Future studies should include more specific measures of mental health, such as clinical scales (eg, PHQ-9) to ascertain new onset psychiatric illness and severity. Adopting rapidly developing techniques that allow for more efficient and bias-corrected estimation using multilevel and other models is also needed.^117,118^ Third, we recognize there are other types of disruptions (eg, COVID-19 deaths of family members)^39^ and COVID-19-related outcomes^119,120^ that may impact mental health and sleep. While our estimates of family financial and school disruptions were designed to correct bias, this does not mean no other relevant disruptions exist. Fourth, we had no information identifying why data were missing. However, we followed best practices^98^ to handle missing data, and results using the hot deck and multiple imputations were consistent, strengthening the validity of our findings.

Nevertheless, our results were likely underestimated as we cannot impute data for nonrespondents to the survey, among which the majority were Black, Hispanic, unmarried, had less than a Bachelor’s degree, and were low-to-middle income. Finally, although this study leveraged longitudinal surveys from May 2020 to December 2020, the ABCD COVID-19-RRR did not collect information immediately after the massive school closure between March 13 to March 15, 2020. We also excluded the last survey as 2021 BLS unemployment rates were not yet linked to ABCD. As the ABCD study is prospectively collecting child health information, future studies can use newer ABCD data releases and link to more structural community-level social determinants of health and policy-related data sources to evaluate the long-term effect of COVID-19 policies and disruptions on child health trajectories.

## Conclusions

These findings suggest that COVID-19 containment policies and geographic variations in unemployment rates may have worsened child mental health, but largely through family financial disruptions and not school disruptions. Therefore, policy makers should consider policies that more effectively mitigate the financial impact of pandemic containment on families and, thereby, child mental well-being.

## Notes

Supplement 1.
eMethods.

eReferences.
**eTable 1.** Recategorization of Oxford COVID-19 Government Response Tracker (OxCGRT) Original Indices to Stringency and Restrictive Policy Index (SRPI) and Support and Flexibility Policy Index (SFPI) **eTable 2.** All measures **eTable 3.** Response and Nonresponse of ABCD COVID Surveys Relative to ABCD Baseline (Surveys 1 through 5) **eTable 4.** Unique Individuals in ABCD Analytic Samples Before Imputation **eTable 5.** First-Stage Estimates for Table 3 **eTable 6.** First-Stage Estimates for Table 4 **eFigure.** Study schema

Supplement 2.
Data Sharing Statement

## References

[1] Johns Hopkins Coronavirus Resource Center. COVID-19 United States cases by county. Johns Hopkins University. Published 2022. Accessed January 16, 2023. https://coronavirus.jhu.edu/us-map

[2] Faulkner J, O’Brien WJ, McGrane B, . Physical activity, mental health and well-being of adults during initial COVID-19 containment strategies: a multi-country cross-sectional analysis. J Sci Med Sport. 2021;24(4):320-326. doi:10.1016/j.jsams.2020.11.016 33341382 PMC7711171

[3] Serrano-Alarcón M, Kentikelenis A, Mckee M, Stuckler D. Impact of COVID-19 lockdowns on mental health: evidence from a quasi-natural experiment in England and Scotland. Health Econ. 2022;31(2):284-296. doi:10.1002/hec.4453 34773325 PMC8646947

[4] Amsalem D, Dixon LB, Neria Y. The coronavirus disease 2019 (COVID-19) outbreak and mental health: current risks and recommended actions. JAMA Psychiatry. 2021;78(1):9-10. doi:10.1001/jamapsychiatry.2020.1730 32579160

[5] Berkowitz SA, Basu S. Unemployment insurance, health-related social needs, health care access, and mental health during the COVID-19 pandemic. JAMA Intern Med. 2021;181(5):699-702. doi:10.1001/jamainternmed.2020.7048 33252615 PMC8094006

[6] Leifheit KM, Pollack CE, Raifman J, . Variation in state-level eviction moratorium protections and mental health among US adults during the COVID-19 pandemic. JAMA Netw Open. 2021;4(12):e2139585. doi:10.1001/jamanetworkopen.2021.39585 34919134 PMC8683968

[7] Wang Y, Shi L, Que J, . The impact of quarantine on mental health status among general population in China during the COVID-19 pandemic. Mol Psychiatry. 2021;26(9):4813-4822. doi:10.1038/s41380-021-01019-y 33483692 PMC7821451

[8] Marroquín B, Vine V, Morgan R. Mental health during the COVID-19 pandemic: effects of stay-at-home policies, social distancing behavior, and social resources. Psychiatry Res. 2020;293:113419. doi:10.1016/j.psychres.2020.113419 32861098 PMC7439968

[9] Blume C, Schmidt MH, Cajochen C. Effects of the COVID-19 lockdown on human sleep and rest-activity rhythms. Curr Biol. 2020;30(14):R795-R797. doi:10.1016/j.cub.2020.06.021 32693067 PMC7284244

[10] Ingram J, Maciejewski G, Hand CJ. Changes in diet, sleep, and physical activity are associated with differences in negative mood during COVID-19 lockdown. Front Psychol. 2020;11:588604. doi:10.3389/fpsyg.2020.588604 32982903 PMC7492645

[11] Dellagiulia A, Lionetti F, Fasolo M, Verderame C, Sperati A, Alessandri G. Early impact of COVID-19 lockdown on children’s sleep: a 4-week longitudinal study. J Clin Sleep Med. 2020;16(9):1639-1640. doi:10.5664/jcsm.8648 32620188 PMC7970607

[12] Parolin Z. Unemployment and child health during COVID-19 in the USA. Lancet Public Health. 2020;5(10):e521-e522. doi:10.1016/S2468-2667(20)30207-3 33007208 PMC7524545

[13] Albrecht JN, Werner H, Rieger N, . Association between homeschooling and adolescent sleep duration and health during COVID-19 pandemic high school closures. JAMA Netw Open. 2022;5(1):e2142100. doi:10.1001/jamanetworkopen.2021.42100 34985517 PMC8733832

[14] Auger KA, Shah SS, Richardson T, . Association between statewide school closure and COVID-19 incidence and mortality in the US. JAMA. 2020;324(9):859-870. doi:10.1001/jama.2020.14348 32745200 PMC7391181

[15] Holmes EA, O’Connor RC, Perry VH, . Multidisciplinary research priorities for the COVID-19 pandemic: a call for action for mental health science. Lancet Psychiatry. 2020;7(6):547-560. doi:10.1016/S2215-0366(20)30168-1 32304649 PMC7159850

[16] Molteni E, Sudre CH, Canas LS, . Illness duration and symptom profile in symptomatic UK school-aged children tested for SARS-CoV-2. Lancet Child Adolesc Health. 2021;5(10):708-718. doi:10.1016/S2352-4642(21)00198-X 34358472 PMC8443448

[17] Dooley DG, Rhodes H, Bandealy A. Pandemic recovery for children-beyond reopening schools. JAMA Pediatr. 2022;176(4):347-348. doi:10.1001/jamapediatrics.2021.3227 35040874

[18] Viner R, Russell S, Saulle R, . School closures during social lockdown and mental health, health behaviors, and well-being among children and adolescents during the first COVID-19 wave: a systematic review. JAMA Pediatr. 2022;176(4):400-409. doi:10.1001/jamapediatrics.2021.5840 35040870

[19] Penninx BWJH, Benros ME, Klein RS, Vinkers CH. How COVID-19 shaped mental health: from infection to pandemic effects. Nat Med. 2022;28(10):2027-2037. doi:10.1038/s41591-022-02028-2 36192553 PMC9711928

[20] Xiao Y, Yip PSF, Pathak J, Mann JJ. Association of social determinants of health and vaccinations with child mental health during the COVID-19 pandemic in the US. JAMA Psychiatry. 2022;79(6):610-621. doi:10.1001/jamapsychiatry.2022.0818 35475851 PMC9047762

[21] Xiao Y, Sharma MM, Thiruvalluru RK, . Trends in psychiatric diagnoses by COVID-19 infection and hospitalization among patients with and without recent clinical psychiatric diagnoses in New York city from March 2020 to August 2021. Transl Psychiatry. 2022;12(1):492. doi:10.1038/s41398-022-02255-8 36414624 PMC9681844

[22] Argabright ST, Tran KT, Visoki E, DiDomenico GE, Moore TM, Barzilay R. COVID-19-related financial strain and adolescent mental health. Lancet Reg Health Am. 2022;16(0):100391. doi:10.1016/j.lana.2022.100391 36405885 PMC9664255

[23] Adegboye D, Williams F, Collishaw S, . Understanding why the COVID-19 pandemic-related lockdown increases mental health difficulties in vulnerable young children. JCPP Adv. 2021;1(1):e12005. doi:10.1111/jcv2.12005 34485985 PMC8250118

[24] Kiss O, Alzueta E, Yuksel D, . The pandemic’s toll on young adolescents: prevention and intervention targets to preserve their mental health. J Adolesc Health. 2022;70(3):387-395. doi:10.1016/j.jadohealth.2021.11.023 35090817 PMC8789404

[25] Yip SW, Jordan A, Kohler RJ, Holmes A, Bzdok D. Multivariate, transgenerational associations of the COVID-19 pandemic across minoritized and marginalized communities. JAMA Psychiatry. 2022;79(4):350-358. doi:10.1001/jamapsychiatry.2021.4331 35138333 PMC8829750

[26] Glied S, Levy H. The potential effects of coronavirus on national health expenditures. JAMA. 2020;323(20):2001-2002. doi:10.1001/jama.2020.6644 32338730

[27] Engelbert Bain L, Berner-Rodoreda A, McMahon SA, . One lesson of COVID-19: conduct more health policy trials. Proc Natl Acad Sci U S A. 2022;119(24):e2119887119. doi:10.1073/pnas.2119887119 35679340 PMC9214500

[28] Fetzer TR, Witte M, Hensel L, . Global Behaviors and Perceptions at the Onset of the COVID-19 Pandemic. National Bureau of Economic Research; 2020. doi:10.3386/w27082

[29] Coibion O, Gorodnichenko Y, Weber M. Labor markets during the COVID-19 crisis: a preliminary view. National Bureau of Economic Research. Accessed February 7, 2023. https://www.nber.org/

[30] Montenovo L, Jiang X, Rojas FL, . Determinants of Disparities in COVID-19 Job Losses. National Bureau of Economic Research; 2020. doi:10.3386/w27132

[31] Brodeur A, Gray D, Islam A, Bhuiyan S. A literature review of the economics of COVID-19. J Econ Surv. 2021;35(4):1007-1044. doi:10.1111/joes.12423 34230772 PMC8250825

[32] Van Lancker W, Parolin Z. COVID-19, school closures, and child poverty: a social crisis in the making. Lancet Public Health. 2020;5(5):e243-e244. doi:10.1016/S2468-2667(20)30084-0 32275858 PMC7141480

[33] How the COVID-19 pandemic has scarred the world’s children. UNICEF. Accessed July 21, 2022. https://www.unicef.org/coronavirus/COVID-19-pandemic-scarred-world-children

[34] Sachs JD, Karim SSA, Aknin L, . The Lancet commission on lessons for the future from the COVID-19 pandemic. Lancet. 2022;400(10359):1224-1280. doi:10.1016/S0140-6736(22)01585-9 36115368 PMC9539542

[35] Lazarus JV, Romero D, Kopka CJ, ; COVID-19 Consensus Statement Panel. A multinational Delphi consensus to end the COVID-19 public health threat. Nature. 2022;611(7935):332-345. doi:10.1038/s41586-022-05398-2 36329272 PMC9646517

[36] CDC Museum COVID-19 Timeline. Centers for Disease Control and Prevention. Accessed July 11, 2022. https://www.cdc.gov/museum/timeline/covid19.html

[37] Hale T, Angrist N, Goldszmidt R, . A global panel database of pandemic policies (Oxford COVID-19 Government Response Tracker). Nat Hum Behav. 2021;5(4):529-538. doi:10.1038/s41562-021-01079-8 33686204

[38] Gassman-Pines A, Ananat EO, Fitz-Henley J II. COVID-19 and parent-child psychological well-being. Pediatrics. 2020;146(4):e2020007294. doi:10.1542/peds.2020-007294 32764151 PMC7546085

[39] Hillis SD, Blenkinsop A, Villaveces A, . COVID-19–associated orphanhood and caregiver death in the United States. Pediatrics. 2021;148(6):e2021053760. doi:10.1542/peds.2021-053760 34620728 PMC10896160

[40] Masonbrink AR, Hurley E. Advocating for children during the COVID-19 school closures. Pediatrics. 2020;146(3):e20201440. doi:10.1542/peds.2020-1440 32554517

[41] Moreno C, Wykes T, Galderisi S, . How mental health care should change as a consequence of the COVID-19 pandemic. Lancet Psychiatry. 2020;7(9):813-824. doi:10.1016/S2215-0366(20)30307-2 32682460 PMC7365642

[42] Fond G, Nemani K, Etchecopar-Etchart D, . Association between mental health disorders and mortality among patients with COVID-19 in 7 countries: a systematic review and meta-analysis. JAMA Psychiatry. 2021;78(11):1208-1217. doi:10.1001/jamapsychiatry.2021.2274 34313711 PMC8317055

[43] Galea S, Merchant RM, Lurie N. The mental health consequences of COVID-19 and physical distancing: the need for prevention and early intervention. JAMA Intern Med. 2020;180(6):817-818. doi:10.1001/jamainternmed.2020.1562 32275292

[44] Raifman J, Green T. Universal masking policies in schools and mitigating the inequitable costs of COVID-19. N Engl J Med. 2022;387:1993-1994. doi:10.1056/NEJMe2213556 36351264 PMC9730911

[45] Sum KK, Cai S, Law E, . COVID-19-related life experiences, outdoor play, and long-term adiposity changes among preschool- and school-aged children in Singapore 1 year after lockdown. JAMA Pediatr. 2022;176(3):280-289. doi:10.1001/jamapediatrics.2021.5585 35072692 PMC8787686

[46] Wang S, Quan L, Chavarro JE, . Associations of depression, anxiety, worry, perceived stress, and loneliness prior to infection with risk of post-COVID-19 conditions. JAMA Psychiatry. 2022;79(11):1081-1091. doi:10.1001/jamapsychiatry.2022.2640 36069885 PMC9453634

[47] Hertz-Palmor N, Moore TM, Gothelf D, . Association among income loss, financial strain and depressive symptoms during COVID-19: evidence from two longitudinal studies. J Affect Disord. 2021;291:1-8. doi:10.1016/j.jad.2021.04.054 34022550 PMC8460400

[48] Yao R, Wu W. Mental disorders associated with COVID-19 related unemployment. Appl Res Qual Life. 2022;17(2):949-970. doi:10.1007/s11482-021-09950-6 33968280 PMC8096626

[49] Badellino H, Gobbo ME, Torres E, . ‘It’s the economy, stupid’: lessons of a longitudinal study of depression in Argentina. Int J Soc Psychiatry. 2022;68(2):384-391. doi:10.1177/0020764021999687 33706611

[50] Koltai J, Raifman J, Bor J, McKee M, Stuckler D. COVID-19 vaccination and mental health: a difference-in-difference analysis of the understanding America study. Am J Prev Med. 2022;62(5):679-687. doi:10.1016/j.amepre.2021.11.006 35012830 PMC8674498

[51] Kessler RC, Chiu WT, Hwang IH, . Changes in prevalence of mental illness among US adults during compared with before the COVID-19 pandemic. Psychiatr Clin North Am. 2022;45(1):1-28. doi:10.1016/j.psc.2021.11.013 35219431 PMC8585610

[52] Wiens KE, Smith CP, Badillo-Goicoechea E, . In-person schooling and associated COVID-19 risk in the United States over spring semester 2021. Sci Adv. 2022;8(16):eabm9128. doi:10.1126/sciadv.abm9128 35442740 PMC9020776

[53] Lessler J, Grabowski MK, Grantz KH, . Household COVID-19 risk and in-person schooling. Science. 2021;372(6546):1092-1097. doi:10.1126/science.abh2939 33927057 PMC8168618

[54] Cowger TL, Murray EJ, Clarke J, . Lifting universal masking in schools—COVID-19 incidence among students and staff. N Engl J Med. 2022;387:1935-1946. doi:10.1056/NEJMoa2211029 36351262 PMC9743802

[55] Chinazzi M, Davis JT, Ajelli M, . The effect of travel restrictions on the spread of the 2019 novel coronavirus (COVID-19) outbreak. Science. 2020;368(6489):395-400. doi:10.1126/science.aba9757 32144116 PMC7164386

[56] Shelleby EC, Pittman LD, Bridgett DJ, Keane J, Zolinski S, Caradec J. Associations between local COVID-19 case rates, pandemic-related financial stress and parent and child functioning. J Fam Psychol. 2022;36(6):932-942. doi:10.1037/fam0000996 35482628

[57] Law EF, Zhou C, Seung F, Perry F, Palermo TM. Longitudinal study of early adaptation to the coronavirus disease pandemic among youth with chronic pain and their parents: effects of direct exposures and economic stress. Pain. 2021;162(7):2132-2144. doi:10.1097/j.pain.0000000000002290 34050112 PMC8205975

[58] Lim MTC, Ramamurthy MB, Aishworiya R, . School closure during the coronavirus disease 2019 (COVID-19) pandemic—impact on children’s sleep. Sleep Med. 2021;78:108-114. doi:10.1016/j.sleep.2020.12.025 33422812 PMC9762095

[59] Esposito S, Principi N. School closure during the coronavirus disease 2019 (COVID-19) pandemic: an effective intervention at the global level? JAMA Pediatr. 2020;174(10):921-922. doi:10.1001/jamapediatrics.2020.1892 32401277

[60] Wooldridge JM. Econometric Analysis of Cross Section and Panel Data. 2nd ed. MIT Press; 2010.

[61] Lipsky AM, Greenland S. Causal directed acyclic graphs. JAMA. 2022;327(11):1083-1084. doi:10.1001/jama.2022.1816 35226050

[62] Garavan H, Bartsch H, Conway K, . Recruiting the ABCD sample: design considerations and procedures. Dev Cogn Neurosci. 2018;32:16-22. doi:10.1016/j.dcn.2018.04.004 29703560 PMC6314286

[63] ABCD Research Consortium. COVID rapid response research (RRR) survey first data release. ABCD Study. Accessed February 7, 2023. 10.15154/1520584

[64] ABCD Research Consortium. COVID Rapid Response Research (RRR) Survey Second Data Release. ABCD Study. Accessed February 7, 2023. 10.15154/1522601

[65] Oxford COVID-19 Government Response Tracker. USA state level COVID-19 Policy Responses. Accessed July 21, 2022. https://github.com/OxCGRT/USA-covid-policy/blob/ba5bda379e8976961c52af9083a90dd2f27da478/data/OxCGRTUS_timeseries_all.xlsx

[66] Cohen S, Kamarck T, Mermelstein R. A global measure of perceived stress. J Health Soc Behav. 1983:385-396. 6668417

[67] Andreou E, Alexopoulos EC, Lionis C, . Perceived stress scale: reliability and validity study in Greece. Int J Environ Res Public Health. 2011;8(8):3287-3298. doi:10.3390/ijerph8083287 21909307 PMC3166743

[68] NIH Toolbox Scoring and Interpretation Guide. National Institutes of Health. Accessed October 11, 2021. https://nihtoolbox.force.com/s/article/nih-toolbox-scoring-and-interpretation-guide

[69] Paolillo EW, McKenna BS, Nowinski CJ, Thomas ML, Malcarne VL, Heaton RK. NIH toolbox emotion batteries for children: factor-based composites and norms. Assessment. 2020;27(3):607-620. doi:10.1177/107319111876639 29618218 PMC6205918

[70] Salsman JM, Butt Z, Pilkonis PA, . Emotion assessment using the NIH Toolbox. Neurology. 2013;80(11)(suppl 3):S76-S86. doi:10.1212/WNL.0b013e3182872e11 23479549 PMC3662334

[71] Pilkonis PA, Choi SW, Salsman JM, . Assessment of self-reported negative affect in the NIH Toolbox. Psychiatry Res. 2013;206(1):88-97. doi:10.1016/j.psychres.2012.09.034 23083918 PMC3561498

[72] Roenneberg T, Wirz-Justice A, Merrow M. Life between clocks: daily temporal patterns of human chronotypes. J Biol Rhythms. 2003;18(1):80-90. doi:10.1177/0748730402239679 12568247

[73] Santisteban JA, Brown TG, Gruber R. Association between the Munich chronotype questionnaire and wrist actigraphy. Sleep Disord. 2018;2018:5646848. doi:10.1155/2018/5646848 29862086 PMC5971234

[74] Afifi L, Kushida CA. Multiple Sleep Latency Test (MSLT). In: Aminoff MJ, Daroff RB, eds. Encyclopedia of the Neurological Sciences. Academic Press; 2003:261-264, doi:10.1016/B0-12-226870-9/00258-6.

[75] Zolovska B, Shatkin JP. Key Differences in pediatric versus adult sleep. In: Kushida CA, ed. Encyclopedia of Sleep. Academic Press; 2013:573-578.

[76] Zolovska B, Shatkin JP. Sleep onset latency. ScienceDirect. Accessed November 26, 2022. https://www.sciencedirect.com/topics/medicine-and-dentistry/sleep-onset-latency

[77] Gropper MA. Avoiding patient harm in anesthesia: human performance and patient safety. ClinicalKey. Accessed November 26, 2022. https://www.clinicalkey.com/#!/content/book/3-s2.0-B9780323596046000067

[78] ScienceDirect. Sleep inertia. ScienceDirect. Accessed November 26, 2022. https://www.sciencedirect.com/topics/medicine-and-dentistry/sleep-inertia

[79] Hirshkowitz M, Whiton K, Albert SM, . National Sleep Foundation’s sleep time duration recommendations: methodology and results summary. Sleep Health. 2015;1(1):40-43. doi:10.1016/j.sleh.2014.12.010 29073412

[80] Paruthi S, Brooks LJ, D’Ambrosio C, . Recommended amount of sleep for pediatric populations: a consensus statement of the American Academy of Sleep Medicine. J Clin Sleep Med. 2016;12(6):785-786. doi:10.5664/jcsm.5866 27250809 PMC4877308

[81] Hallas L, Hale T. Variation in US states’ responses to COVID-19. Blavatnik School of Government. Accessed July 21, 2022. https://www.bsg.ox.ac.uk/research/publications/variation-us-states-responses-covid-19

[82] Stokes J, Turner AJ, Anselmi L, Morciano M, Hone T. The relative effects of nonpharmaceutical interventions on wave one COVID-19 mortality: natural experiment in 130 countries. BMC Public Health. 2022;22(1):1113. doi:10.1186/s12889-022-13546-6 35659646 PMC9165709

[83] Yao H, Wang J, Liu W. Lockdown policies, economic support, and mental health: evidence from the COVID-19 pandemic in United States. Front Public Health. 2022;10:857444. doi:10.3389/fpubh.2022.857444 35719685 PMC9201054

[84] Ochnik D, Rogowska AM, Kuśnierz C, . Mental health prevalence and predictors among university students in nine countries during the COVID-19 pandemic: a cross-national study. Sci Rep. 2021;11(1):18644. doi:10.1038/s41598-021-97697-3 34545120 PMC8452732

[85] Cai W, Zhou Y. Men smoke less under the COVID-19 closure policies: the role of altruism. Soc Sci Med. 2022;306:115159. doi:10.1016/j.socscimed.2022.115159 35753168 PMC9217683

[86] Zaslavsky AM. Exploring potential causal inference through natural experiments. JAMA Health Forum. 2021;2(6):e210289. doi:10.1001/jamahealthforum.2021.0289 36218755

[87] Khullar D, Jena AB. Natural experiments in health care research. JAMA Health Forum. 2021;2(6):e210290. doi:10.1001/jamahealthforum.2021.0290 36218753

[88] Local Area Unemployment Statistics. US Bureau of Labor Statistics. Accessed October 11, 2021. https://www.bls.gov/lau/

[89] Brown TT, Murthy V. Do public health activities pay for themselves: the effect of county-level public health expenditures on county-level public assistance medical care benefits in California. Health Econ. 2020;29(10):1220-1230. doi:10.1002/hec.4130 32618074

[90] Brown TT, Hong JS, Scheffler RM. Evaluating the impact of California’s full service partnership program using a multidimensional measure of outcomes. Adm Policy Ment Health. 2014;41(3):390-400. doi:10.1007/s10488-013-0476-6 23456598

[91] Angrist JD, Pischke JS. Mostly Harmless Econometrics: An Empiricist’s Companion. Princeton University Press; 2009. doi:10.1515/9781400829828

[92] Wooldridge JM. Introductory Econometrics: A Modern Approach. Cengage Learning; 2019.

[93] Cox DR. The Analysis of Multivariate Binary Data. Appl Stat. 1972;21(2):113-120. doi:10.2307/2346482

[94] Wooldridge JM. Control function methods in applied econometrics. J Hum Resour. 2015;50(2):420-445. doi:10.3368/jhr.50.2.420

[95] Abe K, Kawachi I, Watanabe T, Tamiya N. Association of the frequency of in-home care services utilization and the probability of in-home death. JAMA Netw Open. 2021;4(11):e2132787. doi:10.1001/jamanetworkopen.2021.32787 34748009 PMC8576578

[96] Presley CJ, Tang D, Soulos PR, . Association of broad-based genomic sequencing with survival among patients with advanced non-small cell lung cancer in the community oncology setting. JAMA. 2018;320(5):469-477. doi:10.1001/jama.2018.9824 30088010 PMC6142984

[97] Shen YC, Hsia RY. Association between ambulance diversion and survival among patients with acute myocardial infarction. JAMA. 2011;305(23):2440-2447. doi:10.1001/jama.2011.811 21666277 PMC4109302

[98] Little RJA. Statistical Analysis with Missing Data. 3rd ed. John Wiley and Sons, Inc; 2020.

[99] Schonlau M. HOTDECKVAR: Stata module for hotdeck imputation. Accessed December 21, 2022. https://econpapers.repec.org/software/bocbocode/S458527.htm

[100] Heeringa SG, Berglund PA. A guide for population-based analysis of the Adolescent Brain Cognitive Development (ABCD) study baseline data. bioRxiv. Preprint posted online February 10, 2020. doi:10.1101/2020.02.10.942011

[101] Saragosa-Harris NM, Chaku N, MacSweeney N, . A practical guide for researchers and reviewers using the ABCD Study and other large longitudinal datasets. Dev Cogn Neurosci. 2022;55:101115. doi:10.1016/j.dcn.2022.101115 35636343 PMC9156875

[102] Child population by race. Kids Count Data Center. Accessed July 21, 2022. https://datacenter.kidscount.org/data/tables/103-child-population-by-race

[103] Income and poverty in the United States. US Census Bureau. Accessed July 21, 2022. https://www.census.gov/library/publications/2021/demo/p60-273.html

[104] Chakiryan NH, Gore LR, Reich RR, . Survival outcomes associated with cytoreductive nephrectomy in patients with metastatic clear cell renal cell carcinoma. JAMA Netw Open. 2022;5(5):e2212347. doi:10.1001/jamanetworkopen.2022.12347 35576003 PMC9112069

[105] Nenna R, Zeric H, Petrarca L, Mancino E, Midulla F. Weighing policy making: a narrative review of school closures as COVID-19 pandemic-mitigation strategies. Pediatr Pulmonol. 2022;57(9):1982-1989. doi:10.1002/ppul.25787 34894111

[106] Xiong J, Lipsitz O, Nasri F, . Impact of COVID-19 pandemic on mental health in the general population: a systematic review. J Affect Disord. 2020;277:55-64. doi:10.1016/j.jad.2020.08.001 32799105 PMC7413844

[107] Berkowitz SA, Basu S. Unmet social needs and worse mental health after expiration of COVID-19 federal pandemic unemployment compensation. Health Aff (Millwood). 2021;40(3):426-434. doi:10.1377/hlthaff.2020.01990 33600235 PMC8053426

[108] Prime H. Risk and resilience in family well-being during the COVID-19 pandemic. Am Psychol. 2020;75(5):631-643. doi:10.1037/amp0000660 32437181

[109] Rose T, Lindsey MA, Xiao Y, Finigan-Carr NM, Joe S. Mental health and educational experiences among Black youth: a latent class analysis. J Youth Adolesc. 2017;46(11):2321-2340. doi:10.1007/s10964-017-0723-3 28755250

[110] Racine N, McArthur BA, Cooke JE, Eirich R, Zhu J, Madigan S. Global prevalence of depressive and anxiety symptoms in children and adolescents during COVID-19: a meta-analysis. JAMA Pediatr. 2021;175(11):1142-1150. doi:10.1001/jamapediatrics.2021.2482 34369987 PMC8353576

[111] Qin Z, Shi L, Xue Y, . Prevalence and risk factors associated with self-reported psychological distress among children and adolescents during the COVID-19 pandemic in China. JAMA Netw Open. 2021;4(1):e2035487. doi:10.1001/jamanetworkopen.2020.35487 33496797 PMC7838937

[112] Illingworth G, Mansfield KL, Espie CA, Fazel M, Waite F. Sleep in the time of COVID-19: findings from 17000 school-aged children and adolescents in the UK during the first national lockdown. Sleep Adv. 2022;3(1):zpab021. doi:10.1093/sleepadvances/zpab021 35128401 PMC8807290

[113] Yip T, Wang Y, Xie M, Ip PS, Fowle J, Buckhalt J. School start times, sleep, and youth outcomes: a meta-analysis. Pediatrics. 2022;149(6):e2021054068. doi:10.1542/peds.2021-054068 35593065 PMC9665092

[114] Giuntella O, Hyde K, Saccardo S, Sadoff S. Lifestyle and mental health disruptions during COVID-19. Proc Natl Acad Sci U S A. 2021;118(9):e2016632118. doi:10.1073/pnas.2016632118 33571107 PMC7936339

[115] Gurdasani D, Alwan NA, Greenhalgh T, . School reopening without robust COVID-19 mitigation risks accelerating the pandemic. Lancet. 2021;397(10280):1177-1178. doi:10.1016/S0140-6736(21)00622-X 33713595 PMC9755467

[116] Maciejewski ML, Brookhart MA. Using instrumental variables to address bias from unobserved confounders. JAMA. 2019;321(21):2124-2125. doi:10.1001/jama.2019.5646 31046064

[117] Yang Y. Efficient estimation of multi-level models with strictly exogenous explanatory variables. Econ Lett. 2021;198:109667. doi:10.1016/j.econlet.2020.109667

[118] Graham BS, Pinto CC de X. Semiparametrically efficient estimation of the average linear regression function. J Econom. 2022;226(1):115-138. doi:10.1016/j.jeconom.2021.07.008

[119] Martin B, DeWitt PE, Russell S, . Characteristics, outcomes, and severity risk factors associated with SARS-CoV-2 infection among children in the US National COVID Cohort Collaborative. JAMA Netw Open. 2022;5(2):e2143151. doi:10.1001/jamanetworkopen.2021.43151 35133437 PMC8826172

[120] Xiao Y, Sharma MM, Thiruvalluru R, . Trends in psychiatric diagnoses by COVID-19 infection and hospitalization among patients with and without recent clinical psychiatric diagnoses in New York City from March 2020 to August 2021. Transl Psychiatry. Published online November 2022. doi:10.21203/rs.3.rs-1762683/v1 PMC9681844 36414624

