# Supplementary material for: COVID-19 Policies, Pandemic Disruptions, and Changes in Child Mental Health and Sleep in the United States
Source: JAMA Netw Open. 2023 Mar 13;6(3):e232716. doi: 10.1001/jamanetworkopen.2023.2716 (PMC12278772; doi:10.1001/jamanetworkopen.2023.2716)
Supplement: Supplement 1. — eMethods. eReferences. eTable 1. Recategorization of Oxford COVID-19 Government Response Tracker (OxCGRT) Original Indices to Stringency and Restrictive Policy Index (SRPI) and Support and Flexibility Policy Index (SFPI) eTable 2. All measures eTable 3. Response and Nonresponse of ABCD COVID Surveys Relative to ABCD Baseline (Surveys 1 through 5) eTable 4. Unique Individuals in ABCD Analytic Samples Before Imputation eTable 5. First-Stage Estimates for Table 3 eTable 6. First-Stage Estimates for Table 4 eFigure. Study schema [file jamanetwopen-e232716-s001.pdf]

Supplemental Online Content

Xiao Y, Brown TT, Snowden LR, Chow JCC, Mann JJ. COVID-19 policies, pandemic disruptions, and changes in child mental health and sleep in the United States. *JAMA Netw Open*. 2023;6(3):e232716. doi:10.1001/jamanetworkopen.2023.2716

eMethods.

eReferences.

eTable 1. Recategorization of Oxford COVID-19 Government Response Tracker (OxCGRT) Original Indices to Stringency and Restrictive Policy Index (SRPI) and Support and Flexibility Policy Index (SFPI)

eTable 2. All Measures

eTable 3. Response and Non-Response of ABCD COVID Surveys Relative to ABCD Baseline (Surveys 1 through 5)

eTable 4. Unique Individuals in ABCD Analytic Samples Before Imputation

eTable 5. First-Stage Estimates for Table 3

eTable 6. First-Stage Estimates for Table 4

eFigure. Study schema

This supplemental material has been provided by the authors to give readers additional information about their work.

© 2023 Xiao Y et al. *JAMA Network Open.*

eMethods.

1. ABCD COVID Rapid Response Research (RRR) Survey

The Adolescent Brain Cognitive Development Study℠ (ABCD Study®), the largest longitudinal study of brain development and child health in the United States, follows 11,878 children over 10 years. Children and parents were recruited from 21 U.S. research sites, at ages 9-10, in 2016-18. In March 2020, the world became substantially affected by the COVID-19 pandemic, leading to an upheaval in the economy and the lives of almost every family. The ABCD Study developed brief surveys sent electronically to all ABCD participants and their participating parent/guardian about the impact of the pandemic on their lives. An overview of the ABCD Study is at https://abcdstudy.org.

From 2020 to 2021, the ABCD Rapid Response Research (RRR) sent six surveys. The ABCD RRR surveys were disseminated on May 16-22, 2020 (n = 7240, Survey 1), June 24-27, 2020 (n = 7554, Survey 2), August 4-5, 2020 (n = 6,852, Survey 3), October 8, 2020 (n = 6,688, Survey 4), December 13, 2020 (n = 6,068, Survey 5), and March 2, 2021 (n = 5,929, Survey 6). We used surveys 1-5 in this study to be consistent with the geocoded measures of county-level COVID-19 infection rates and unemployment rates (see eMethod 3). We merged the data from the first and second releases of the ABCD COVID RRR to the ABCD baseline from the ABCD main study in order to merge sociodemographic characteristics).

Child and parent characteristics of non-responders and overall response rates are presented in eTable 3.

© 2023 Xiao Y et al. *JAMA Network Open.*

2. Measurements for sleep in ABCD COVID-19 Rapid Research Release

Questions about typical sleep behavior in the past week are derived from the Munich Chronotype Questionnaire (Roenneberg et al. Life between clocks: daily temporal patterns of human chronotypes. *J Biol Rhythms*, 2003, 18: 80-90); however, the time period is for the past week only, and no distinction is made in the ABCD COVID-19 Rapid Research Release surveys between school days and school-free days (e.g., weekends). Time resolution is hours for bedtime and sleep time (night-time hour options only), wake-up time, and school start time (daytime hour options only). Variables available are:

- Time of going to bed (time resolution in hours; night-time hours only)
- Time of actually starting to fall asleep (time resolution in hours; night-time hour options only)
- Minutes needed to fall asleep
- Number of awakenings during the night (up to 10)
- Total time awake during the night
- Time of waking up (time resolution in hours; daytime hour options only)
- Time of starting school work (time resolution in hours; daytime hour options only)

From the responses, we computed four dimensions of sleep continuity:

1. Sleep Latency (mins)
   a. Continuous version: Time from turning the light off to falling asleep (mctq_fd_min_to_sleep_cv, asking "Report on your typical sleep behavior over the past week. : I need _________ minutes to fall asleep."), which we recalibrated the responses to minutes.
   b. Categorical version: We also calculated pathological/abnormal sleep latency, which is a binary variable categorizing children whose sleep latency is out of the normal range between 10–26 minutes.[1–3]
2. Sleep Inertia (mins)
   a. Continuous version: Time taken to get out of bed (mctq_fd_min_to_get_up_cv, asking "Report on your typical sleep behavior over the past week. : After _________ minutes I get up."), which we recalibrated the responses to minutes as the time resolution.
   b. Categorical version: We also calculated pathological/abnormal sleep inertia, which is a binary variable categorizing children whose sleep inertia is out of the normal range between 15–30 minutes.[4,5]
3. Sleep Duration (hours)
   a. Continuous version: Calculated using "sleep ends" subtracted by "sleep onset".
   b. "Sleep ends" is the time resolution (in hours) of waking up (mctq_fd_wake_up_time_cv, asking "Report on your typical sleep behavior over the past week. : I wake up at:").
   c. "Sleep onset" is calculated by adding time (in hours) of actually starting to fall asleep (mctq_fd_time_asleep_cv, Report on your typical sleep behavior over the past week. : I actually start trying to fall asleep at:) to time (in hours) of going to bed (mctq_fd_btime_cv, asking "Report on your typical sleep behavior over the past week. : I go to bed at:").
   d. Categorical version: We also calculated pathological/abnormal sleep duration, a binary variable categorizing children with insufficient sleep duration under 8 hours, which was the recommended amount of sleep by both the National Sleep Foundation and the American Academy of Sleep Medicine.[6,7]

The exact variable names extracted from the ABCD raw data can be found in eTable 2.

© 2023 Xiao Y et al. *JAMA Network Open.*

3. Additional data sources for geocoded social determinants of health (SDoH) variables and policy index

Geocoded data linked to each child were available in the COVID Rapid Response Research (RRR) Second Releases.[8,9] These data describe the local environment for each participant. COVID-19 new cases per 100,000 population are from the John Hopkins University (JHU), and unemployment rates are from the U.S. Bureau of Labor Statistics at the county level.

JHU COVID-19 data sources are cited on their README.md and found at https://github.com/CSSEGISandData/COVID-19. COVID-19 death counts (cumulative) were used to calculate new case/death counts and aggregated rolling 7-day averages. Metrics were population adjusted by US Census measures per county: https://www.census.gov/data/datasets/time-series/demo/popest/2010s-counties-total.html

Unemployment rates are provided at the county level monthly by the U.S. Bureau of Labor Statistics: https://www.bls.gov/lau/. The rates are not seasonally adjusted. These county-level unemployment data are also available for 2019 (i.e., pre-pandemic) compared to 2020/2021. However, the BLS-ABCD data linkage for 2021 was unavailable at the time of the manuscript preparation. Therefore, survey 6 was omitted.

The policy index data were from the US sub-national policy data[10] from the Oxford COVID-19 Government Response Tracker (OxCGRT). We linked state-level policy indexes to ABCD families through the states they resided in during COVID-19.[11] The OxCGRT includes extensive data on COVID-19-related policies and converts these data into indexes.[12] The policy indexes reflect variation in state responses over time and by region and identify correlates of more or less intense responses. We recategorized the original indexes into two new indexes: the Stringency and Restrictive Policy Index (SRPI) and the Support and Flexibility Policy Index (SFPI).

All data sources, constructs, variables, and measures are summarized in eTable 2.

© 2023 Xiao Y et al. *JAMA Network Open.*

4. Instrumental variable approach to remove biases

We performed instrumental variable analyses to address bias from unobserved confounders, measurement error, and reverse causation (simultaneity).[13] Instrumental variables analyses incorporate the use of exogenous external factors (external factors whose values cannot be affected by the individual children and parents we are examining) whose impact on outcomes only works through the endogenous exposures of interest (schooling disruption and family financial disruption), conditional on the included covariates.[14]

Our instruments, Stringency and Restriction Policy Index (SRPI), Support and Flexibility Policy Index (SFPI), and the unemployment rate, are necessarily exogenous to any individual family/child. Since the outcome measures we examine are for children, who are unlikely to be aware of the SRPI, SRFI, or the US Bureau of Labor Statistics (BLS) unemployment rates, it is reasonable that these instruments would only impact children through noticeable impacts on the two direct disruptions on children's daily life these instruments will correlate with: school disruptions and family financial disruption. We do not control for anything on the causal pathway between family financial disruption, school disruption, and child mental health and sleep outcomes.

However, for our instruments to be valid, they must not correlate with anything in the second-stage error term. Thus, any potential factors in the second-stage error term that the instruments may be correlated with, apart from family financial disruptions and school disruptions, must be conditioned out of the second-stage error term by being included in the second-stage equation as a covariate.

There are two important factors that the instruments will be correlated with that therefore must be included in the second-stage equation to avoid correlation of the instruments with the second-stage error term. First, although many children will be unaware of local COVID policy and unemployment rates, these may attract news reports and, thus, child awareness. To avoid the correlation of the SRPI, SRFI, and unemployment rates with news media information in the second-stage error term, we include child exposure to news media as a covariate in the second-stage equation.

Similarly, new COVID-19 cases are directly affected by the SRPI, SRFI, and local unemployment rates. Thus, new COVID-19 cases must be eliminated from the second-stage error term by including them as a covariate in the second-stage equation.

We estimated our models using two-stage limited information maximum likelihood (2SLIML) linear regression models for continuous outcomes and 2SLIML linear probability models for binary outcomes. Linear probability models yield virtually identical marginal effects as logistic regression when outcomes are within [0.2, 0.8][15] and allow us to perform the appropriate weak instrument tests and incorporate multiple binary endogenous variables. To determine the joint strength of our instruments, we perform Stock-Yogo weak instrument tests using 2SLIML.[16] 2SLIML models are median unbiased and allow for somewhat weaker instruments than identically specified two-stage least squares models without inducing bias.[17]

Clustering at the site level is included because while the exposures of interest (financial disruption and schooling disruption) vary within sites, our instruments only vary across sites and time. We also weight our results to closely approximate the distribution of individual characteristics in the American Community Survey.[18] All analyses were performed in Stata 16.

© 2023 Xiao Y et al. *JAMA Network Open.*

eReferences

1. Afifi L, Kushida CA. Multiple Sleep Latency Test (MSLT). In: Aminoff MJ, Daroff RB, eds. *Encyclopedia of the Neurological Sciences*. Academic Press; 2003:261-264. doi:10.1016/B0-12-226870-9/00258-6

2. Zolovska B, Shatkin JP. Key Differences in Pediatric versus Adult Sleep. In: Kushida CA, ed. *Encyclopedia of Sleep*. Academic Press; 2013:573-578. doi:10.1016/B978-0-12-378610-4.00496-4

3. ScienceDirect. Sleep Onset Latency - an overview | ScienceDirect Topics. Published 2022. Accessed November 26, 2022. https://www.sciencedirect.com/topics/medicine-and-dentistry/sleep-onset-latency

4. Gropper MA. Avoiding Patient Harm in Anesthesia: Human Performance and Patient Safety - ClinicalKey. Published 2020. Accessed November 26, 2022. https://www.clinicalkey.com/#!/content/book/3-s2.0-B9780323596046000067

5. ScienceDirect. Sleep Inertia - an overview | ScienceDirect Topics. Published 2022. Accessed November 26, 2022. https://www.sciencedirect.com/topics/medicine-and-dentistry/sleep-inertia

6. Hirshkowitz M, Whiton K, Albert SM, et al. National Sleep Foundation's sleep time duration recommendations: methodology and results summary. *Sleep Health*. 2015;1(1):40-43. doi:10.1016/j.sleh.2014.12.010

7. Paruthi S, Brooks LJ, D 'Ambrosio Carolyn, et al. Recommended Amount of Sleep for Pediatric Populations: A Consensus Statement of the American Academy of Sleep Medicine. *J Clin Sleep Med*. 2016;12(06):785-786. doi:10.5664/jcsm.5866

8. ABCD Research Consortium. *COVID Rapid Response Research (RRR) Survey First Data Release*.; 2020. http://dx.doi.org/12805210.15154/1520584

9. ABCD Research Consortium. *COVID Rapid Response Research (RRR) Survey Second Data Release*.; 2021. http://dx.doi.org/10.15154/1522601

10. Hallas L, Hale T. Variation in US states' responses to COVID-19 | Blavatnik School of Government. Published May 7, 2021. Accessed July 21, 2022. https://www.bsg.ox.ac.uk/research/publications/variation-us-states-responses-covid-19

11. Oxford Covid-19 Government Response Tracker. USA state level Covid-19 Policy Responses. Published online July 21, 2022. Accessed July 21, 2022. https://github.com/OxCGRT/USA-covid-policy/blob/ba5bda379e8976961c52af9083a90dd2f27da478/data/OxCGRTUS_timeseries_all.xlsx

12. Hale T, Angrist N, Goldszmidt R, et al. A global panel database of pandemic policies (Oxford COVID-19 Government Response Tracker). *Nat Hum Behav*. 2021;5(4):529-538. doi:10.1038/s41562-021-01079-8

13. Maciejewski ML, Brookhart MA. Using Instrumental Variables to Address Bias From Unobserved Confounders. *JAMA*. 2019;321(21):2124. doi:10.1001/jama.2019.5646

14. Wooldridge JM. *Econometric Analysis of Cross Section and Panel Data, Second Edition*. MIT Press; 2010.

15. Cox DR. The Analysis of Multivariate Binary Data. *J R Stat Soc Ser C Appl Stat*. 1972;21(2):113-120. doi:10.2307/2346482

16. Stock J, Yogo M. Testing for Weak Instruments in Linear IV Regression. In: *Identification and Inference for Econometric Models*. Cambridge University Press; 2005:80-108. Accessed April 8, 2021. https://econpapers.repec.org/paper/nbrnberte/0284.htm

17. Angrist JD, Pischke JS. *Mostly Harmless Econometrics: An Empiricist's Companion*. Princeton University Press; 2008. doi:10.2307/j.ctvcm4j72

18. US Census. American Community Survey 1-Year Data (2005-2021). Census.gov. Published 2022. Accessed December 18, 2022. https://www.census.gov/data/developers/data-sets/acs-1year.html

© 2023 Xiao Y et al. *JAMA Network Open.*

eTable 1. Recategorization of Oxford COVID-19 Government Response Tracker (OxCGRT) Original Indices to Stringency and Restrictive Policy Index (SRPI) and Support and Flexibility Policy Index (SFPI)[a]

	OxCGRT Original Categories		
	Stringency index	Economic support index	Containment and health index
**Stringency and Restrictive Policy Index (SRPI)**			
School Closing	C-1		C-1
Workplace Closing	C-2		C-2
Cancel Public Events	C-3		C-3
Restrictions on Gatherings	C-4		C-4
Close Public Transport	C-5		C-5
Stay-at-Home Requirements	C-6		C-6
Restrictions on Local Movement	C-7		C-7
Restrictions on International Travel	C-8		C-8
Public Information Campaigns	H-1		H-1
**Support and Flexibility Policy Index (SFPI)**			
Income Support		E-1	
Debt Relief		E-2	
Testing Policy			H-2
Contact Tracing			H-3
Masking			H-6
Vaccination Policy			H-7
Protection of Elderly			H-8

[a.] Primary OxCGRT data for countries is available in https://github.com/OxCGRT/covid-policy-tracker/tree/master/data; USA state level Covid-19 Policy Responses is available in https://github.com/OxCGRT/USA-covid-policy. There are slight differences between primary OxCGRT data and the US data, where the US subnational data contains policies issued by the US federal government (NAT_GOV) and policies issued by state-level governments and sub-state governments (STATE_WIDE). OxCGRT data contains 21 indicators and a miscellaneous notes field organized into five groups: C - containment and closure policies; E - economic policies; H - health system policies; V - vaccination policies; M - miscellaneous policies. We recategorized the original domains and indices into two main categories: 1) stringency and restriction index (SRI), which records the strictness of 'lockdown style' closure and containment policies that primarily restrict people's behaviors; 2) support and flexibility index (SFI), which reflect how much economic support has been made available (such as income support and debt relief) and how many efforts have been made to protect citizen health with health measures that do entail significant travel restrictions (such as testing policies, contact tracing, protection of elderly people, and mask mandates).
Cite as: Laura Hallas, Ariq Hatibie, Saptarshi Majumdar, Monika Pyarali, Rachelle Koch, Andrew Wood and Thomas Hale (2020). [Variation in US states' responses to COVID-19_3.0] (https://www.bsg.ox.ac.uk/research/publications/variation-us-states-responses-covid-19). Blavatnik School of Government

eTable 2. All Measures

Construct	Data Sources	Element Name	Original Question/Definitions	Response Categories	Recoding Rules (new variables)
**Study Design Variables**					
Personal ID	ABCD	src_subject_id			
Family ID	ABCD	rel_family_id			
Study Site ID	ABCD	site_id_l			
**Time Variables**					
Survey wave	ABCD	eventname			
**Outcome: Mental Health**					
Perceived Stress	ABCD	pstr_confidence_p_cv	In the last month, how often have you felt confident about your ability to handle your personal problems?	0 = Never; 1 = Almost Never; 2 = Sometimes; 3 = Fairly often; 4 = Very Often	Summed Score
	ABCD	pstr_overcome_p_cv	In the last month, how often have you felt difficulties were piling up so high that you could not overcome them?	0 = Never; 1 = Almost Never; 2 = Sometimes; 3 = Fairly often; 4 = Very Often	
	ABCD	pstr_unable_control_cv	In the last month, how often have you felt that you were unable to control the important things in your life?	0 = Never; 1 = Almost Never; 2 = Sometimes; 3 = Fairly often; 4 = Very Often	
	ABCD	pstr_way_p_cv	In the last month, how often have you felt that things were going your way?	0 = Never; 1 = Almost Never; 2 = Sometimes; 3 = Fairly often; 4 = Very Often	

© 2023 Xiao Y et al. *JAMA Network Open.*

Construct	Data Sources	Element Name	Original Question/Definitions	Response Categories	Recoding Rules (new variables)
NIH Toolbox (sadness)	ABCD	felt_alone_cv	In the past week. . . : I felt alone	1 = Never; 2 = Almost Never; 3 = Sometimes; 4 = Often; 5 = Almost Always	Summed Score
		felt_always_sad	In the past week. . . : I could not stop feeling sad		
		felt_cv	In the past week. . . : I felt like I couldn't do anything right		
		felt_life_went_wrong_cv	In the past week. . . : I felt everything in my life went wrong		
		felt_lonely_cv	In the past week. . . : I felt lonely		
		felt_no_fun_cv	It was hard for me to have fun		
		felt_sad_cv	In the past week. . . : I felt sad		
		felt_unhappy_cv	In the past week. . . : I felt unhappy		

© 2023 Xiao Y et al. *JAMA Network Open.*

Construct	Data Sources	Element Name	Original Question/Definitions	Response Categories	Recoding Rules (new variables)
NIH Toolbox (positive affect)	ABCD	attentive_y_cv	Please rate how each item describes you now or within the past week: I felt attentive (that is, alert or able to pay attention)	1 = Not true; 3 = Somewhat true; 5 = Very true; 777 = Refuse to answer	Summed Score
		calm_y_cv	Please rate how each item describes you now or within the past week. I felt calm		
		concentrate_y_cv	Please rate how each item describes you now or within the past week. I felt able to concentrate		
		confident_y_cv	Please rate how each item describes you now or within the past week. I felt confident		
		delighted_y_cv	Please rate how each item describes you now or within the past week. I felt delighted		
		ease_y_cv	Please rate how each item describes you now or within the past week. I felt at ease (Definition: relaxed, comfortable)		
		energetic_y_cv	Please rate how each item describes you now or within the past week. I felt energetic		
		enthusiastic_y_cv	Please rate how each item describes you now or within the past week. I felt enthusiastic (Definition: very excited)		
		interested_y_cv	Please rate how each item describes you now or within the past week. I felt interested		

© 2023 Xiao Y et al. *JAMA Network Open.*

Construct	Data Sources	Element Name	Original Question/Definitions	Response Categories	Recoding Rules (new variables)
COVID-19 Worry	ABCD	worry_y_cv	In the past week...How worried have you been about coronavirus (COVID-19)?	1 = Not at all; 2 = Slightly; 3 = Moderately; 4 = Very; 5 = Extremely	
**Outcome: Sleep (recoded)**					
Sleep Latency (mins)	ABCD	mctq_slat	Time from turning the light off to falling asleep		
Local time of preparing to go to sleep (24 hh:mm)	ABCD	mctq_sprep	Local time of pareparing to go to bed		
Sleep onset (24 hh:mm)	ABCD	mctq_so	Local time of falling asleep		mctq_sprep + mctq_slat
Sleep Inertia (mins)	ABCD	mctq_sine	Time taken to get out of bed		
Local time of getting out of bed	ABCD	mctq_sgu	Local time of getting out of bed		mctq_se + mctq_sine
Sleep Duration (24 hh:mm)	ABCD	mctq_sdu	Sleep end subtracted by sleep onset		mctq_se - mctq_so
Local time of going to bed (24 hh:mm)	ABCD	mctq_sbt	Local time of going to bed		
Mid-Sleep (24 hh:mm)	ABCD	mctq_msf	Mid-point between sleep onset and sleep end		mctq_so + mctq_sdu/2
**Outcome: Sleep (original)**					
Total time awake during the night	ABCD	mctq_fd_awakening_min_cv	Report on your typical sleep behavior over the past week. : Altogether, these awakenings last ______minutes.	0 = 0 ; 1 = 1 ; 2 = 2 ; 3 = 3 ; 4 = 4 ; 5 = 5 ; 6 = 6 ; 7 = 7 ; 8 = 8 ; 9 = 9 ; 10 = 10 ; 11 = 15 ; 12 = 20 ; 13 = 25 ; 14 = 30 ; 15 = 40 ; 16 = 50 ; 17 = 1 hour ; 18 = 1 hour 15 minute ; 19 = 1 hour 30 minute ; 20 = 1 hour 45 minute ; 21 = 2 hours ; 22 = 3 hours ; 23 = 4 hours [mctq_fd_num_wake_up_cv] > 0""	

© 2023 Xiao Y et al. *JAMA Network Open.*

Construct	Data Sources	Element Name	Original Question/Definitions	Response Categories	Recoding Rules (new variables)
Time of going to bed	ABCD	mctq_fd_btime_cv	Report on your typical sleep behavior over the past week. : I go to bed at:	13 = 6 PM; 1 = 7 PM ; 2 = 8 PM ; 14=8:30 PM; 3 = 9 PM ; 15=9:30 PM ; 4 = 10 PM ; 16=10:30 PM; 5 = 11 PM ; 6 = 12 AM ; 7 = 1 AM ; 8 = 2 AM ; 9 = 3 AM ; 10 = 4 AM ; 11 = 5 AM ; 12 = 6 AM	mctq_sbt
Minutes needed to get up	ABCD	mctq_fd_min_to_get_up_cv	Report on your typical sleep behavior over the past week. : After _________ minutes I get up.	0 = 0 ; 1 = 1 ; 2 = 2 ; 3 = 3 ; 4 = 4 ; 5 = 5 ; 6 = 6 ; 7 = 7 ; 8 = 8 ; 9 = 9 ; 10 = 10 ; 11 = 15 ; 12 = 20 ; 13 = 25 ; 14 = 30 ; 15 = 40 ; 16 = 50 ; 17 = 1 hour ; 18 = 1 hour 15 minute ; 19 = 1 hour 30 minute ; 20 = 1 hour 45 minute ; 21 = 2 hours ; 22 = 3 hours ; 23 = 4 hours	mctq_sine
Minutes needed to fall asleep	ABCD	mctq_fd_min_to_sleep_cv	Report on your typical sleep behavior over the past week. : I need _________ minutes to fall asleep.	0 = 0 ; 1 = 1 ; 2 = 2 ; 3 = 3 ; 4 = 4 ; 5 = 5 ; 6 = 6 ; 7 = 7 ; 8 = 8 ; 9 = 9 ; 10 = 10 ; 11 = 15 ; 12 = 20 ; 13 = 25 ; 14 = 30 ; 15 = 40 ; 16 = 50 ; 17 = 1 hour ; 18 = 1 hour 15 minute ; 19 = 1 hour 30 minute ; 20 = 1 hour 45 minute ; 21 = 2 hours ; 22 = 3 hours ; 23 = 4 hours	mctq_slat
Number of awakenings during the night	ABCD	mctq_fd_num_wake_up_cv	Report on your typical sleep behavior over the past week. : After falling asleep, I wake up ____ times during the night.	0 = 0 ; 1 = 1 ; 2 = 2 ; 3 = 3 ; 4 = 4 ; 5 = 5 ; 6 = 6 ; 7 = 7 ; 8 = 8 ; 9 = 9 ; 10 = 10	

© 2023 Xiao Y et al. *JAMA Network Open.*

Construct	Data Sources	Element Name	Original Question/Definitions	Response Categories	Recoding Rules (new variables)
Time of actually starting to fall asleep	ABCD	mctq_fd_time_asleep_cv	Report on your typical sleep behavior over the past week. : I actually start trying to fall asleep at:	13 = 6 PM; 1 = 7 PM ; 2 = 8 PM ; 14=8:30 PM; 3 = 9 PM ; 15=9:30 PM ; 4 = 10 PM ; 16=10:30 PM; 5 = 11 PM ; 6 = 12 AM ; 7 = 1 AM ; 8 = 2 AM ; 9 = 3 AM ; 10 = 4 AM ; 11 = 5 AM ; 12 = 6 AM	mctq_sprep
Time of waking up	ABCD	mctq_fd_wake_up_time_cv	Report on your typical sleep behavior over the past week. : I wake up at:	1 = 4 AM ; 15=4:30 AM ;2 = 5 AM ; 16=5:30 AM; 3=6 AM ; 17= 6:30 AM; 4 = 7 AM ; 18= 7:30 AM; 5 = 8 AM ; 19= 8:30 AM; 6 = 9 AM ; 20 = 9:30 AM ;7 =10 AM ; 21=10:30 AM ; 8 = 11 AM ; 9 = 12 PM ; 10 = 1 PM ; 11 = 2 PM ; 12 = 3 PM ; 14 = 4 PM	mctq_se
Time of starting school work	ABCD	mctq_schedule_time_cv	Report on your typical sleep behavior over the past week. : I start my school work at:	1 = 4 AM ; 16=4:30 AM; 2 = 5 AM ; 17=5:30 AM; 3=6 AM ; 18=6:30 AM; 4 = 7 AM ; 19= 7:30 AM ; 5 = 8 AM ; 20 = 8:30 AM; 6 = 9 AM ; 21= 9:30 AM ;7 =10 AM ; 22=10:30 AM; 8 = 11 AM ; 9 = 12 PM ; 10 = 1 PM ; 11 = 2 PM ; 12 = 3 PM ; 14 = 4 PM ; 15 = Not applicable	
**Exposure Variable: COVID-related Disruptions**					
Financial Disruptions	ABCD	fam_wage_loss_cv	Since January 2020 has anyone in your household lost wages sales or work due to the impact of coronavirus on employment business or the economy?	1 = No ; 2 = Yes ; 777 = Refuse to answer	

© 2023 Xiao Y et al. *JAMA Network Open.*

Construct	Data Sources	Element Name	Original Question/Definitions	Response Categories	Recoding Rules (new variables)
School Disruptions	ABCD	school_at_home_cv<br>school_close_cv<br>school_close_date_cv	Is your child currently doing some or all of their school-at-home?<br>Has your child's school been closed physically due to coronavirus?<br>Last date of in-person class		
**Instrumental Variable: Policy Index**					
Stringency and Restriction Policy Index (SRPI)	Oxford COVID-19 Government Response Tracker (OxCGRT)		See eTable 1		
Support and Flexibility Policy Index (SFPI)	Oxford COVID-19 Government Response Tracker (OxCGRT)		See eTable 1		
**Instrumental Variable: Local Unemployment Rates**					
County-level Unemployment	ABCD	covidgeo_bls_unemployment2020	Bureau of Labor Statistics 2020 monthly unemployment rate (not seasonally adjusted) county-level		
**Covariates: Children sociodemographic characteristics**					

© 2023 Xiao Y et al. *JAMA Network Open.*

Construct	Data Sources	Element Name	Original Question/Definitions	Response Categories	Recoding Rules (new variables)
Race/Ethnicity	ABCD	race_ethnicity		White<br>Black<br>Hispanic<br>Asian<br>Multi-Racial	
Income	ABCD	demo_comb_income_v2		<50K & >=50K<br>>100K	
Sex	ABCD	sex		0 = Male; 1 = Female	
Age	ABCD	interview_age		Computed from months to years of the age, and categorized into two groups: 0 = 8-9; 1 = 10-11	
Parental education	ABCD	demo_prnt_ed_p<br>demo_prtnr_ed_p		>=Bachelor<br>< Bachelor	
Marital status of the parents	ABCD	demo_prnt_marital_p		Married<br>Widowed<br>Divorced<br>Separated<br>Never married<br>Living with partner	
Child social media exposure	ABCD	child_news_time_cv	Over the past week about how much time per day do you think your child has been getting news from television news sources about the coronavirus and its impact? (hours)		
County-level new COVID-19 cases	ABCD	covidgeo_jhu_newcase100k_7_db0	Johns Hopkins Covid-19 new case count normalized by population (per 100k) 7-day rolling mean. Same day as questionnaire dissemination date		

© 2023 Xiao Y et al. *JAMA Network Open.*

eTable 3. Response and Nonresponse of ABCD COVID Surveys Relative to ABCD Baseline (Surveys 1 through 5)

Characteristic	Survey 1	Survey 2	Survey 3	Survey 4	Survey 5
Child Characteristics					
Female, Count (%)					
Non-Response	1891 (35.3%)	1773 (33.1%)	2069 (38.7%)	2135 (39.9%)	2435 (45.5%)
Response	3462 (64.7%)	3580 (66.9%)	3284 (61.3%)	3218 (60.1%)	2918 (54.5%)
Male, Count (%)					
Non-Response	2213 (37.7%)	2013 (34.3%)	2402 (40.9%)	2509 (42.7%)	2809 (47.8%)
Response	3659 (62.3%)	3859 (65.7%)	3470 (59.1%)	3363 (57.3%)	3063 (52.2%)
Asian, Count (%)					
Non-Response	59 (24.5%)	58 (24.1%)	72 (29.9%)	76 (31.5%)	75 (31.1%)
Response	182 (75.5%)	183 (75.9%)	169 (70.1%)	165 (68.5%)	166 (68.9%)
Black, Count (%)					
Non-Response	873 (54.7%)	814 (51.0%)	920 (57.6%)	947 (59.3%)	1035 (64.8%)
Response	724 (45.3%)	783 (49.0%)	677 (42.4%)	650 (40.7%)	562 (35.2%)
Hispanic, Count (%)					
Non-Response	952 (42.8%)	943 (42.4%)	1079 (48.6%)	1092 (49.1%)	1199 (54.0%)
Response	1270 (57.2%)	1279 (57.6%)	1143 (51.4%)	1130 (50.9%)	1023 (46.0%)
Other Race, Count (%)					
Non-Response	423 (36.0%)	372 (31.7%)	461 (39.2%)	501 (42.6%)	569 (48.4%)
Response	752 (64.0%)	803 (68.3%)	714 (60.8%)	674 (57.4%)	606 (51.6%)
White, Count (%)					
Non-Response	1794 (30.0%)	1596 (26.7%)	1937 (32.4%)	2026 (33.9%)	2365 (39.5%)
Response	4189 (70.0%)	4387 (73.3%)	4046 (67.6%)	3957 (66.1%)	3618 (60.5%)
Parental Characteristics					
Married, Count (%)					
Non-Response	2362 (30.8%)	2185 (28.5%)	2585 (33.7%)	2736 (35.6%)	3143 (40.9%)
Response	5314 (69.2%)	5491 (71.5%)	5091 (66.3%)	4940 (64.4%)	4533 (59.1%)

© 2023 Xiao Y et al. *JAMA Network Open.*

Characteristic	Survey 1	Survey 2	Survey 3	Survey 4	Survey 5
Unmarried, Count (%)					
Non-Response	1697 (48.9%)	1553 (44.7%)	1830 (52.7%)	1851 (53.3%)	2044 (58.9%)
Response	1775 (51.1%)	1919 (55.3%)	1642 (47.3%)	1621 (46.7%)	1428 (41.1%)
<Bachelor's Degree, Count (%)					
Non-Response	2189 (49.9%)	2068 (47.2%)	2383 (54.4%)	2395 (54.6%)	2619 (59.7%)
Response	2195 (50.1%)	2316 (52.8%)	2001 (45.6%)	1989 (45.4%)	1765 (40.3%)
Bachelor's Degree or higher, Count (%)					
Non-Response	1913 (28.0%)	1714 (25.1%)	2084 (30.5%)	2244 (32.9%)	2621 (38.4%)
Response	4916 (72.0%)	5115 (74.9%)	4745 (69.5%)	4585 (67.1%)	4208 (61.6%)
High Income, Count (%)[a]					
Non-Response	1221 (27.5%)	1143 (25.8%)	1370 (30.9%)	1484 (33.5%)	1700 (38.3%)
Response	3213 (72.5%)	3291 (74.2%)	3064 (69.1%)	2950 (66.5%)	2734 (61.7%)
Low-Middle Income, Count (%)[a]					
Non-Response	2452 (41.7%)	2203 (37.5%)	2619 (44.6%)	2666 (45.4%)	3016 (51.3%)
Response	3422 (58.3%)	3671 (62.5%)	3255 (55.4%)	3208 (54.6%)	2858 (48.7%)

© 2023 Xiao Y et al. *JAMA Network Open.*

eTable 4. Unique Individuals in ABCD Analytic Samples Before Imputation

	Mental Health Sample (N=6030)	Sleep Sample (N=6080)
Child Characteristics		
Biological Sex: Frequency (%)		
Female, Count (%)	2947 (48.9%)	2968 (48.8%)
Male, Count (%)	3083 (51.1%)	3112 (51.2%)
Race/Ethnicity: Frequency (%)		
Asian, Count (%)	273 (4.5%)	273 (4.5%)
Black, Count (%)	461 (7.6%)	470 (7.7%)
Hispanic, Count (%)	1167 (19.4%)	1174 (19.3%)
Other Race, Count (%)	347 (5.7%)	350 (5.8%)
White, Count (%)	3783 (62.7%)	3814 (62.7%)
Parental Characteristics: Frequency (%)		
Married, Count (%)	4285 (71.1%)	4320 (71.1%)
Unmarried, Count (%)	1745 (28.9%)	1760 (28.9%)
>=Bachelor's Degree, Count (%)	2096 (34.8%)	2116 (34.8%)
<Bachelor's Degree, Count (%)	3934 (65.2%)	3964 (65.2%)
Income >$100 000, Count (%)	2184 (36.2%)	2203 (36.2%)
Income <$50 000 or $50 000-$100 000, Count (%)	3846 (63.8%)	3877 (63.8%)

Note: Weighted to the American Community Survey

© 2023 Xiao Y et al. *JAMA Network Open.*

eTable 5. First-Stage Estimates for Table 3

Characteristics	(1)	(2)	(5)	(6)	(7)	(8)
	Perceived Stress COVID-19 Worry		Sadness		Positive Affect	
	School Disruption First Stage	Financial Disruption First Stage	School Disruption First Stage	Financial Disruption First Stage	School Disruption First Stage	Financial Disruption First Stage
Child Characteristics						
Age (years)	-0.017	-1.118	-0.219	-1.420[c]	0.373	-1.103
	(-0.864, 0.829)	(-2.303, 0.066)	(-0.989, 0.550)	(-2.592, -0.248)	(-1.176, 1.923)	(-2.420, 0.215)
Female	-2.376[a]	-0.498	-1.678[c]	-0.197	-3.265[b]	-0.208
	(-3.764, -0.988)	(-3.227, 2.231)	(-3.057, -0.299)	(-3.236, 2.842)	(-5.669, -0.861)	(-2.629, 2.213)
Asian	6.280[a]	-5.704[c]	7.985[a]	-6.860[b]	3.999	-3.441
	(2.969, 9.592)	(-10.422, -0.986)	(3.924, 12.046)	(-11.550, -2.170)	(-2.010, 10.008)	(-9.497, 2.615)
Black	15.950[a]	-1.596	13.122[a]	-0.986	19.842[a]	-2.419
	(12.862, 19.038)	(-5.528, 2.337)	(10.049, 16.195)	(-6.451, 4.479)	(16.317, 23.367)	(-7.540, 2.703)
Hispanic	8.141[a]	2.656	8.349[a]	2.640	6.955[a]	2.586
	(6.169, 10.112)	(-0.628, 5.940)	(5.946, 10.751)	(-1.008, 6.288)	(3.790, 10.119)	(-1.529, 6.701)
Other Race	6.126[a]	-0.213	5.625[b]	-0.368	7.431[a]	-1.221
	(3.365, 8.887)	(-6.820, 6.394)	(2.087, 9.164)	(-7.584, 6.847)	(4.230, 10.633)	(-8.858, 6.416)
Parental Characteristics						
>=Bachelor's Degree	-2.932[c]	-13.302[a]	-2.634	-13.884[a]	-2.497	-13.677[a]
	(-5.381, -0.482)	(-16.786, -9.818)	(-5.476, 0.208)	(-17.964, -9.805)	(-5.504, 0.510)	(-16.995, -10.360)
<$100 000 per year	-0.515	5.124[c]	-0.221	4.166	-0.874	6.661b
	(-2.394, 1.365)	(0.785, 9.462)	(-2.165, 1.724)	(-0.164, 8.495)	(-3.378, 1.630)	(1.836, 11.486)
Not Married	1.240	-3.204	0.547	-2.746	2.120	-4.146[c]
	(-0.580, 3.061)	(-6.491, 0.084)	(-0.817, 1.912)	(-6.490, 0.998)	(-1.506, 5.747)	(-8.089, -0.202)
Environmental Factors (COVID)						
Child Media Exposure (hours)	0.040	-0.075	-0.026	-0.059	0.146	-0.105
	(-0.070, 0.150)	(-0.234, 0.085)	(-0.148, 0.096)	(-0.255, 0.136)	(-0.023, 0.315)	(-0.361, 0.150)
COVID-19 Incidence/100,000	0.009	0.039	0.012	0.032	0.000	0.032

© 2023 Xiao Y et al. *JAMA Network Open.*

Characteristics	(1)	(2)	(5)	(6)	(7)	(8)
	(-0.064, 0.082)	(-0.007, 0.085)	(-0.070, 0.095)	(-0.026, 0.089)	(-0.080, 0.081)	(-0.036, 0.100)
Instruments						
SRPI (natural log)	33.211[a]	-1.477	41.216[a]	-1.993	23.418[b]	-6.242
	(18.089, 48.333)	(-8.954, 6.000)	(21.587, 60.844)	(-10.639, 6.654)	(6.854, 39.983)	(-18.911, 6.428)
SFPI (natural log)	13.291[c]	10.216[b]	7.702	11.040[b]	23.440[b]	10.477[c]
	(1.609, 24.973)	(3.880, 16.551)	(-6.302, 21.706)	(3.729, 18.350)	(9.423, 37.457)	(0.763, 20.190)
BLS Unemployment Rate	0.361[b]	0.505[a]	0.498[a]	0.371[b]	0.222	0.650[a]
	(0.125, 0.596)	(0.312, 0.697)	(0.218, 0.778)	(0.097, 0.645)	(-0.082, 0.526)	(0.400, 0.900)
Survey 2	-46.918[a]	0.434			-53.841[a]	-1.323
	(-52.766, -41.069)	(-1.900, 2.768)			(-58.543, -49.138)	(-4.525, 1.879)
Survey 3	-51.283[a]	1.243	-51.047[a]	0.880		
	(-58.568, -43.998)	(-1.243, 3.728)	(-57.810, -44.283)	(-1.636, 3.397)		
Survey 4	5.042	1.530				
	(-0.333, 10.417)	(-1.107, 4.167)				
Survey 5	1.487	0.504	2.246	0.550		
	(-4.698, 7.671)	(-2.702, 3.710)	(-3.867, 8.359)	(-3.192, 4.291)		
Constant	-94.671[a]	40.930[b]	-112.445a	46.913[b]	-79.966[c]	59.219[b]
	(-148.47, -40.87)	(11.903, 69.958)	(-175.61, -49.28)	(13.667, 80.159)	(-144.17, -15.76)	(22.967, 95.471)
Observations (Individuals)	25,555 (8400)	25,555 (8400)	14,610 (7447)	14,610 (7447)	9,408 (6302)	9,408 (6302)
Weak Instrument Test for LIML (Joint test of both first-stage results)						
Kleibergen-Paap rk Wald F Statistic	10.49		6.54		10.08	
Stock-Yogo LIML Critical Value (10%)	5.44		5.44		5.44	

Robust 95% CI in parentheses

[a] p≤0.001, [b] p≤0.01, [c] p≤0.05

© 2023 Xiao Y et al. *JAMA Network Open.*

eTable 6. First-Stage Estimates for Table 4

Characteristics	(1)	(2)
	Sleep Latency (Abnormal)<br>Sleep Inertia (Abnormal)<br>Sleep Duration (Abnormal)	
	School Disruption First Stage	Financial Disruption First Stage
Child Characteristics		
Age (years)	-0.053	-0.012[c]
	(-0.887, 0.782)	(-0.024 - -0.000)
Female	-2.253[b]	-0.005
	(-3.696, -0.810)	(-0.033 - 0.022)
Asian	6.299[a]	-0.057[c]
	(2.985, 9.613)	(-0.104 - -0.011)
Black	16.004[a]	-0.015
	(12.862, 19.146)	(-0.053 - 0.024)
Hispanic	7.846[a]	0.028
	(5.936, 9.755)	(-0.006 - 0.061)
Other Race	6.184[a]	-0.001
	(3.475, 8.893)	(-0.065 - 0.063)
Parental Characteristics		
>=Bachelor's Degree	-2.961[c]	-0.132[a]
	(-5.341, -0.582)	(-0.167 - -0.096)
<$100 000 per year	-0.462	0.055[c]
	(-2.320, 1.396)	(0.012 - 0.097)
Not Married	1.314	-0.033[c]
	(-0.451, 3.079)	(-0.066 - -0.001)
Environmental Factors (COVID)		
Child Media Exposure (hours)	0.030	-0.001

© 2023 Xiao Y et al. *JAMA Network Open.*

Characteristics	(1)	(2)
	(-0.078, 0.139)	(-0.002 - 0.001)
COVID-19 Incidence/100,000	0.011	0.000
	(-0.061, 0.084)	(-0.000 - 0.001)
Instruments		
SRPI (natural log)	33.482[a]	-0.018
	(18.613, 48.352)	(-0.095 - 0.058)
SFPI (natural log)	13.494[c]	0.101[b]
	(2.097, 24.891)	(0.036 - 0.166)
BLS Unemployment Rate	0.377[b]	0.005[a]
	(0.145, 0.609)	(0.003 - 0.007)
Survey 5	1.593	0.004
	(-4.605, 7.791)	(-0.028 - 0.037)
Survey 2	-46.600[a]	0.006
	(-52.394, -40.806)	(-0.018 - 0.029)
Survey 3	-50.910[a]	0.014
	(-58.193, -43.626)	(-0.012 - 0.039)
Survey 4	5.462[c]	0.015
	(0.004, 10.919)	(-0.013 - 0.043)
Survey 5	1.593	0.004
	(-4.605, 7.791)	(-0.028 - 0.037)
Observations (Individuals)	25,948 (8472)	25,948 (8472
Weak Instrument Test for LIML (Joint test of both first-stage results)		
Kleibergen-Paap rk Wald F Statistic	11.32	
Stock-Yogo LIML Critical Value (10%)	5.44	

Robust 95% CI in parentheses [a] p≤0.001, [b] p≤0.01, [c] p≤0.05

© 2023 Xiao Y et al. *JAMA Network Open.*

eFigure. Study Schema

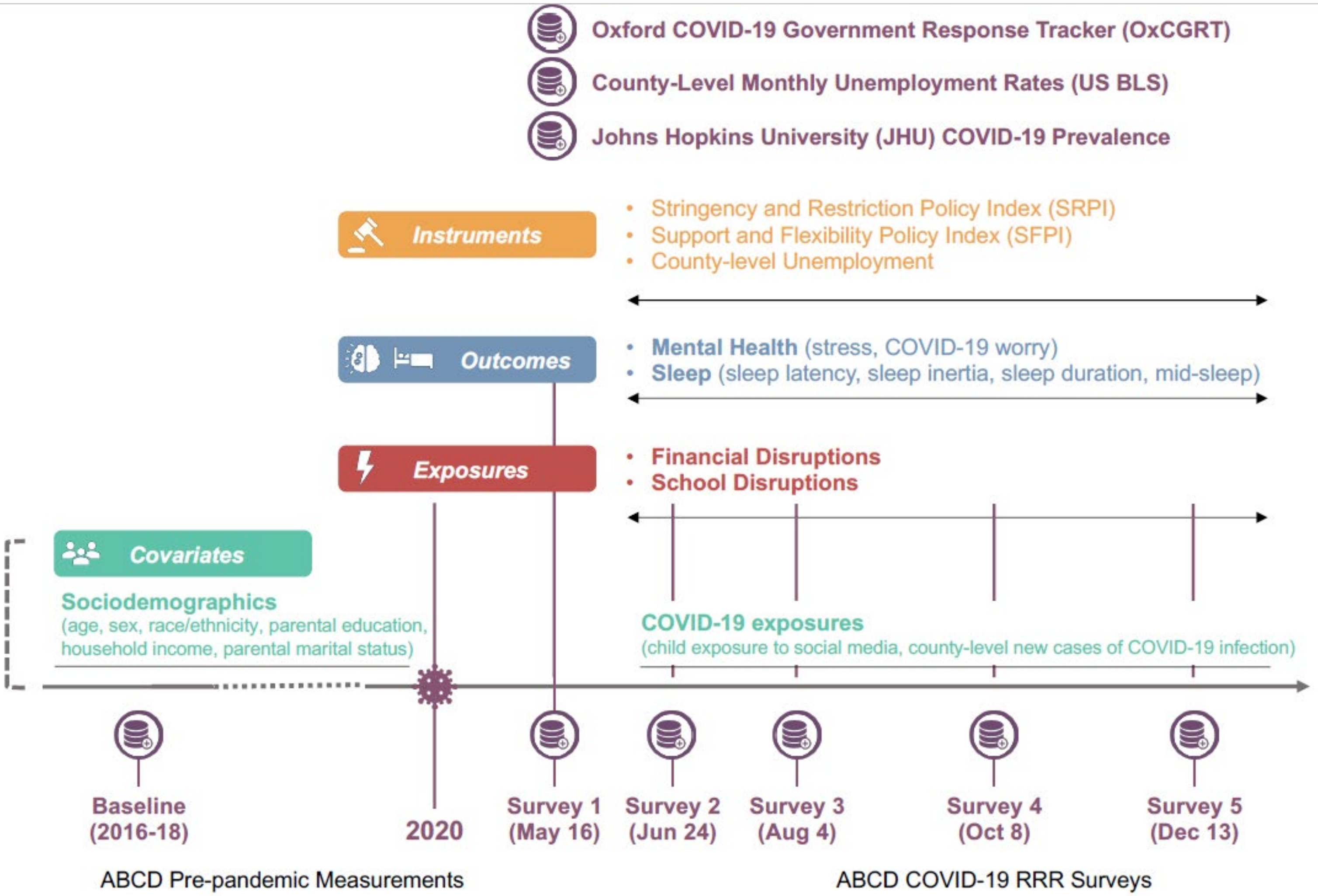

© 2023 Xiao Y et al. *JAMA Network Open.*
