# Supplementary material for: COVID-19 Policies, Pandemic Disruptions, and Changes in Child Mental Health and Sleep in the United States
Source: JAMA Netw Open. 2023 Mar 13;6(3):e232716. doi: 10.1001/jamanetworkopen.2023.2716 (PMC12278772; doi:10.1001/jamanetworkopen.2023.2716)
Supplement: Supplement 2. — Data Sharing Statement [file jamanetwopen-e232716-s002.pdf]

Data Sharing Statement

Xiao. COVID-19 Policies, Pandemic Disruptions, and Changes in Child Mental Health and Sleep in the United States. *JAMA Netw Open*. Published March 13, 2023. doi:10.1001/jamanetworkopen.2023.2716

Data
Data available: No

Additional Information
Explanation for why data not available: All data from the current study comes from the Adolescent Brain and Cognitive Development (ABCD) study. Because of data use agreement restrictions we were not able to make data freely available. However, data can be accessed with approval of the ABCD consortium at https://nda.nih.gov/abcd/request-access. The data used in the current manuscript do not require any special permissions and are available to all authorized users of ABCD study data.
